# Gold-activated persulfate p-doping of organic semiconductors

**DOI:** 10.1038/s41563-026-02547-0

**Published:** 2026-03-17

**Authors:** Tiefeng Liu, Matilde Silveri, Zesheng Liu, Sang Young Jeong, Qiao He, Giannis G. Gkikas, Wenlong Jin, Chi-Yuan Yang, Tom P. A. van der Pol, Feng Zhang, Christina Kousseff, Anna Martinelli, Iain McCulloch, Martin Heeney, Han Young Woo, Alessandro Motta, Mats Fahlman, Simone Fabiano

**Affiliations:** 1 Laboratory of Organic Electronics, Department of Science and Technology, Linköping University, Norrköping, Sweden; 2 Wallenberg Initiative Materials Science for Sustainability, Department of Science and Technology, Linköping University, Norrköping, Sweden; 3 Department of Chemistry, College of Science, Korea University, Seoul, Republic of Korea; 4 College of Education Sciences, The Hong Kong University of Science and Technology (Guangzhou), Guangzhou, China; 5 Department of Chemistry and Chemical Engineering, Chalmers University of Technology, Gothenburg, Sweden; 6 Department of Chemistry, University of Oxford, Oxford, UK; 7 Andlinger Center for Energy and the Environment and Department of Electrical and Computer Engineering, Princeton University, Princeton, NJ USA; 8 Physical Sciences and Engineering Division, King Abdullah University of Science and Technology, Thuwal, Kingdom of Saudi Arabia; 9 Dipartimento di Scienze Chimiche, Università di Roma ‘La Sapienza’ and INSTM, UdR Roma, Rome, Italy

**Keywords:** Electronic devices, Electron transfer

## Abstract

Chemical doping is crucial for fine-tuning the electronic properties of organic semiconductors (OSCs) and enhancing device performance across various technologies. While several methods for controlled dopant distribution have been explored, achieving lateral doping gradients via simple solution processing remains challenging. Here we present a gold-activated persulfate doping strategy in which persulfate is catalytically activated at gold surfaces to generate SO_4_^•−^ radicals that locally oxidize (p-dope) the OSCs. This reaction creates a lateral doping gradient extending outwards from the gold interface, as verified by spectroscopic and electrical characterization. The approach is broadly applicable to OSCs spanning a 1.5-eV ionization potential range and yields conductivities >1,900 S cm^−1^. To demonstrate the impact of this method, we applied gold-activated persulfate doping to modulate contact regions in solution-processed organic field-effect transistors, achieving reduced contact resistance and improved charge-carrier mobility. This simple, scalable approach enables the formation of lateral doping gradients from solution and offers new opportunities for interfacial tuning in organic electronics.

## Abstract (web summary)

Achieving lateral doping gradients in organic semiconductors (OSCs) via solution processing is crucial but remains a challenge. A gold-activated persulfate doping strategy can locally oxidize OSCs and create a lateral doping gradient, enabling low contact resistance and high carrier mobility in solution-processed organic field-effect transistors.

## Main

Chemical doping is a key process in organic semiconductors (OSCs), used to tailor their electronic properties^1,2^ and enhance device performance in applications such as solar cells^3,4^, light-emitting diodes^5,6^, thermoelectrics^7,8^, transistors^9,10^ and electrochemical systems^11,12^. Advances in dopant design and doping strategies have enabled conductivities exceeding 10^3^ S cm^−1^ (refs. ^13–17^). Conventional doping methods typically involve either coprocessing (for example, co-blending in solution or co-evaporation) or sequential deposition (for example, spin-coating or vapour-depositing the dopant onto a preformed OSC film), both of which generally lead to uniformly doped films^18^. However, the ability to locally dope specific regions of OSCs via wet processing remains an open challenge, with major implications for enhancing device performance across a wide range of technologies, including solution-processed organic field-effect transistors (OFETs)^19,20^, organic electrochemical transistors^21,22^, organic thermoelectric generators^23^ and other applications where precise doping of the OSC/contact regions is critical for optimal operation^24,25^.

Various strategies for spatially modulating dopant distribution have been explored, including laser patterning^26,27^, dopant deposition via thermal evaporation^28,29^ or printing^30,31^, light-assisted doping^32,33^ and electrochemical ion implantation^34^. While effective, these approaches often rely on energy-intensive or technically demanding conditions, limiting their scalability. By contrast, achieving spatial modulation of doping levels through solution processing remains difficult. Although vertical doping gradients have been demonstrated using kinetically controlled doping in solution with bulky molecular dopants^35^, achieving lateral gradients in dopant distribution via solution processing remains an unresolved challenge.

**Fig. 1:**
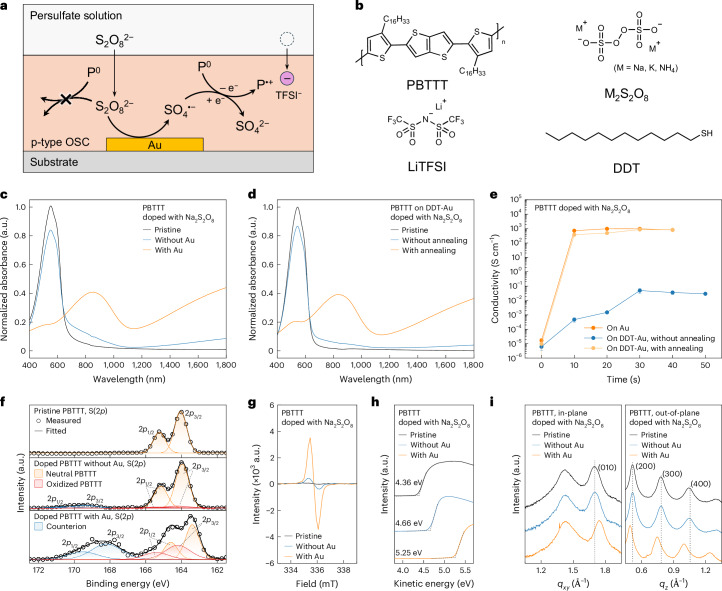
GAP doping of PBTTT. **a**,**b**, Schematic illustration of the GAP doping method (**a**), along with the chemical structures of the persulfate dopant, LiTFSI counterion, PBTTT and DDT (**b**). **c**, Absorption spectra of PBTTT films deposited on bare glass and gold-coated glass substrates after doping with Na_2_S_2_O_8_. **d**, Absorption spectra of PBTTT films deposited on DDT-modified gold substrates upon doping with Na_2_S_2_O_8_ before and after thermal annealing at 180 °C, a process that causes deprotection of the gold surface. **e**, Electrical conductivity of PBTTT films measured using bare gold or DDT-modified gold electrode after doping with Na_2_S_2_O_8_. Points represent mean values; error bars show standard deviation (s.d., not visible at this scale); *n* = 10 independent samples. **f**, S(2*p*) XPS spectra of undoped and persulfate-doped PBTTT films with and without gold. **g**, EPR spectra of undoped and persulfate-doped PBTTT films with and without gold. **h**, UPS spectra of undoped and persulfate-doped PBTTT films with and without gold. **i**, GIWAXS line cuts of undoped and persulfate-doped PBTTT films with and without gold. Source data

Here, we introduce a gold-activated persulfate (GAP) doping strategy that enables the formation of lateral doping gradients in OSCs from solution. We show that persulfate is catalytically activated upon contact with gold, generating SO_4_^•−^ radicals that strongly oxidize (p-dope) OSCs, as schematically illustrated in Fig. 1a. This localized activation leads to a lateral doping gradient, as confirmed by spatially resolved optical spectroscopy. The method is applicable to a variety of p-type OSCs with ionization potentials (IPs) spanning a 1.5-eV range and achieves electrical conductivities exceeding 1,900 S cm^−1^. To demonstrate the potential of this approach, we used GAP to modulate the OSC’s doping level at the source/drain contact regions in OFETs, reducing contact resistance by ~10× at low gate bias and doubling carrier mobility. By enabling simple, scalable formation of lateral doping gradients from solution, this approach offers new opportunities for interfacial tuning and performance enhancement in next-generation organic electronic devices.

## GAP doping of PBTTT

Sodium persulfate (Na_2_S_2_O_8_; Fig. 1b) is a potent and air-stable oxidizing agent, commonly used for soil and groundwater remediation^36^, as well as a polymerization initiator in the synthesis of both commodity^37^ and conjugated^38^ polymers. However, when poly(2,5-bis(3-hexadecylthiophen-2-yl)thieno[3,2-*b*]thiophene) (PBTTT) films deposited on glass substrates were immersed in a 10-mM Na_2_S_2_O_8_ solution, only minimal doping was observed. The ultraviolet–visible–near-infrared spectra showed a slight reduction in the neutral absorption peak at ~550 nm and a modest increase in polaron absorption in the 700–1,000-nm region (Fig. 1c). By contrast, PBTTT films deposited on gold-coated glass and exposed to the same doping conditions exhibited complete bleaching of the neutral peak and a strong polaron absorption band centred around ~820 nm, extending into the infrared region (Fig. 1c; see optical photographs in Supplementary Fig. 1). Quartz crystal microbalance with dissipation monitoring (QCM-D) measurements confirmed that persulfate solution penetrates PBTTT films (Supplementary Fig. 2), indicating that the limited doping on glass is not due to poor diffusion but rather to the low oxidation efficiency of Na_2_S_2_O_8_ alone, consistent with previous reports that used persulfate with other polymer systems in the absence of gold^39,40^. Passivating the gold surface with a self-assembled monolayer of 1-dodecanethiol (DDT) hindered the emergence of the polaron bands in the absorption spectra (Fig. 1d). However, when subjected to thermal annealing at 180 °C, a process known to cause desorption of DDT from gold^41^, the samples exhibited full bleaching of the neutral peak and strong polaron absorption at wavelengths above 650 nm (Fig. 1d). These observations indicate that a clean gold surface plays a critical role in enabling the doping process. Other metals commonly used in organic electronic devices, such as chromium and copper, also supported GAP doping, while aluminium and silver showed only minimal effects (Supplementary Fig. 3), probably due to surface reactions (oxidation, passivation and/or leaching) that interfere with sustained radical generation at the metal–OSC interface.

**Extended Data Fig. 1:**
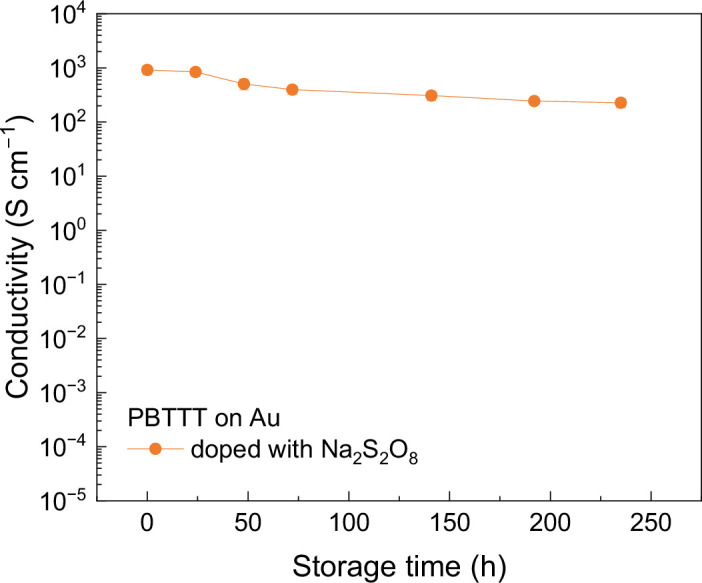
Ambient stability of GAP doped PBTTT. Electrical conductivity of PBTTT films doped with Na_2_S_2_O_8_ in the presence of LiTFSI, measured over extended storage under ambient laboratory conditions (≈22 °C, ∼40% RH). The conductivity remained well above 200 S cm^−1^ after 235 h in air. Points represent mean values; error bars show standard deviation (s.d., not visible at this scale); n = 10 independent samples. Source data

**Extended Data Fig. 2:**
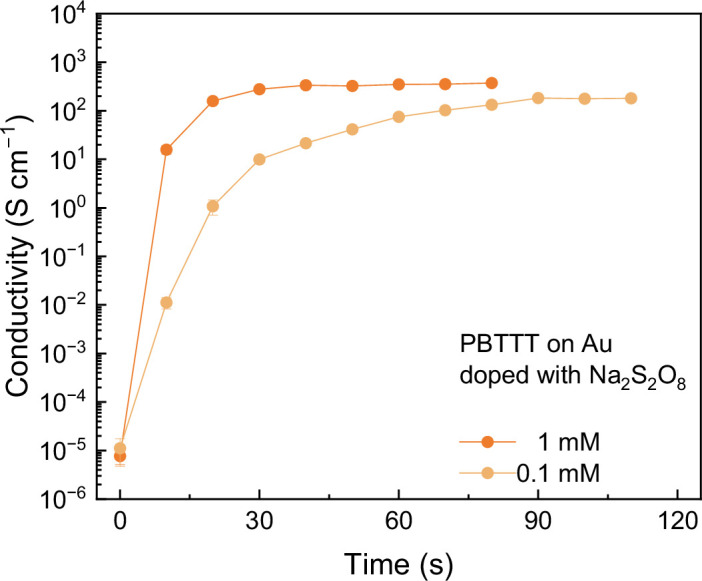
Effect of persulfate concentration. Electrical conductivity of PBTTT films measured on bare gold electrodes after doping with Na_2_S_2_O_8_ in the presence of LiTFSI as the counterion. Points represent mean values; error bars show standard deviation (s.d., not visible at this scale); n = 10 independent samples. Source data

In parallel with the optical changes, a substantial difference in electrical conductivity was observed when comparing bare gold electrodes with either DDT-modified gold or indium tin oxide (ITO) electrodes (Fig. 1e). PBTTT films with bare gold electrodes showed a rapid increase in conductivity within 10 s, reaching a maximum of 965 ± 59 S cm^−1^ after approximately 30 s, consistent with the rapid colour change observed in Supplementary Video 1. Note that the conductivity remained well above 200 S cm^−1^ even after 235 h of storage under ambient conditions (Extended Data Fig. 1). Lower dopant concentrations led to slower doping kinetics, as shown in Extended Data Fig. 2. For comparison, PBTTT films doped using F_4_TCNQ/ion exchange reached a substantially lower maximum conductivity of 563 ± 39 S cm^−1^ (Supplementary Fig. 4), a value consistent with previous reports^42^. By contrast, persulfate-doped PBTTT films using ITO or DDT-modified gold electrodes exhibited conductivities three to four orders of magnitude lower (0.40 ± 0.19 S cm^−1^ for ITO and 0.049 ± 0.018 S cm^−1^ for DDT-modified gold; Fig. 1e and Supplementary Fig. 5). It should be noted that the lower conductivity observed for PBTTT on DDT-modified gold, compared with that on ITO, is probably due to the absence of thermal annealing, which was deliberately avoided to prevent DDT desorption. Indeed, annealing removes the DDT layer, reactivates the gold surface and restores conductivity to levels comparable to those of unmodified gold electrodes (Fig. 1e).

Other persulfate salts, such as potassium persulfate (K_2_S_2_O_8_) and ammonium persulfate ((NH_4_)_2_S_2_O_8_), were also able to dope PBTTT in the presence of bare gold electrodes, yielding conductivities of 653 ± 37 and 611 ± 22 S cm^−1^, respectively (Supplementary Fig. 6). All electrical conductivity measurements were performed in the presence of lithium bis(trifluoromethanesulfonyl)imide (LiTFSI), dissolved in the same acetonitrile solution, with TFSI^−^ ions serving as counterions to balance the positive charge on the PBTTT chains. LiTFSI itself has no doping ability, as confirmed by negligible changes in absorbance and conductivity (Supplementary Fig. 7).

**Extended Data Fig. 3:**
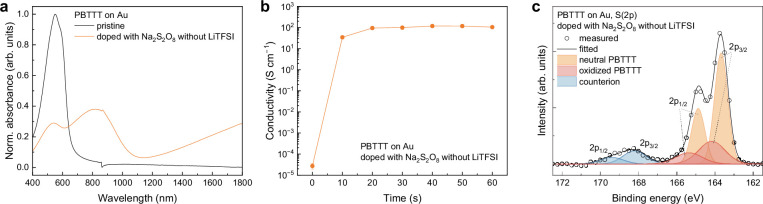
GAP doping in the absence of counterions. **a**, Absorption spectra and **b**, electrical conductivity of PBTTT films on gold doped with Na_2_S_2_O_8_ in the absence of LiTFSI. Points represent mean values; error bars show standard deviation (s.d., not visible at this scale); n = 10 independent samples. **c**, XPS S(2p) spectrum of PBTTT on gold doped with Na_2_S_2_O_8_ without LiTFSI. The doping level is estimated to be ≈29% from the ratio of the oxidized PBTTT signal to the total PBTTT S(2p) area. Source data

**Extended Data Fig. 4:**
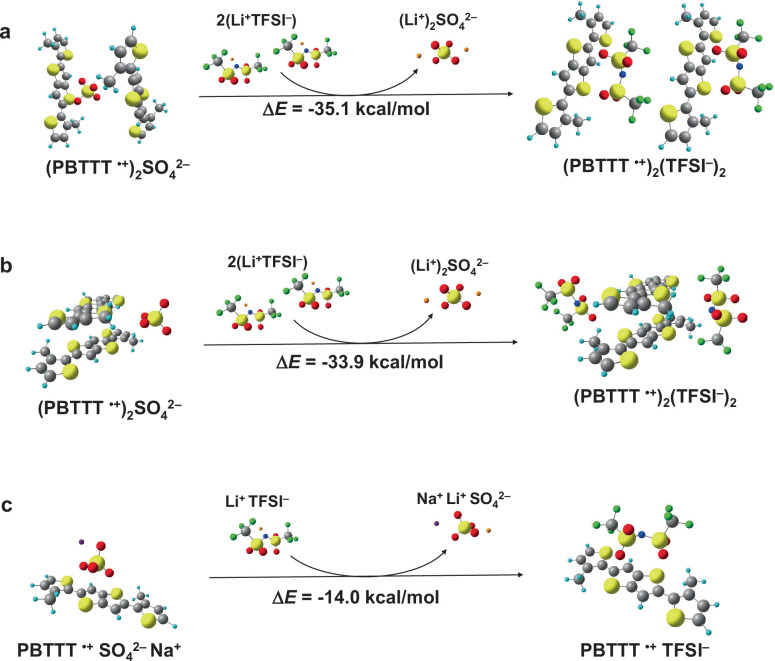
Energetics of counterion exchange in doped PBTTT. Ion exchange process between (PBTTT^•+^)_2_SO_4_^2‒^ species and Li^+^TFSI^‒^. The ion-exchange energetics were calculated using DFT (M06 hybrid functional with the 6–311 + G^**^ basis set) as implemented in Gaussian 16. Geometry optimizations were performed without constraints, and solvent effects (acetonitrile) were included using the PCM model. Conformations of (PBTTT^•+^)_2_SO_4_^2‒^ and PBTTT^•+^TFSI^‒^ were taken from literature (see Ref. ^42^). Three conformations were considered: (**a**) counterion intercalated between two PBTTT^•+^ molecules, (**b**) counterion located on the lateral side of two stacked PBTTT^•+^ molecules, and (**c**) ion exchange involving only a single PBTTT^•+^ molecule. In all cases, replacement of SO_4_^2‒^ by TFSI^‒^ was found to be exothermic, in agreement with the experimental results.

**Extended Data Fig. 5:**
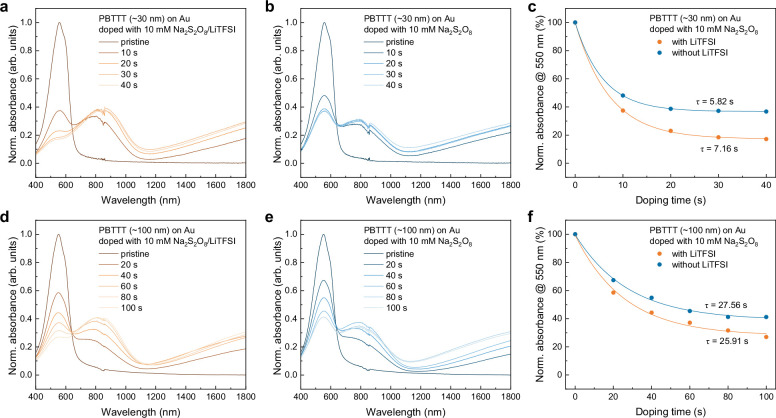
Kinetics of GAP doping with and without LiTFSI. **a**, Absorbance evolution of thin PBTTT films (~30 nm) doped with Na_2_S_2_O_8_ in the presence of LiTFSI, and (**b**) corresponding evolution without LiTFSI. **c**, Decay fitting of the neutral peak absorbance for the thin films. **d**, Absorbance evolution of thick PBTTT films (~100 nm) doped with Na_2_S_2_O_8_ in the presence of LiTFSI, and (**e**) corresponding evolution without LiTFSI. **f**, Decay fitting of the neutral peak absorbance for the thick films. In both thin and thick films, the neutral peak exhibits similar decay times under Na_2_S_2_O_8_ doping with or without LiTFSI, indicating that LiTFSI does not affect the doping kinetics. Source data

Even in the absence of LiTFSI, PBTTT films doped with Na_2_S_2_O_8_ on bare gold electrodes still reached relatively high doping levels, as indicated by bleaching of the neutral peak, emergence of the polaron band and conductivities exceeding 100 S cm^−1^ (Extended Data Fig. 3). The addition of LiTFSI, however, increases conductivity by nearly an order of magnitude. LiTFSI does not induce doping but promotes a spontaneous SO_4_^2–^/TFSI^−^ counterion-exchange process, as supported by density functional theory (DFT) calculations, which show the exchange to be exothermic and therefore thermodynamically favourable (Extended Data Fig. 4), consistent with earlier observation of rapid anion exchange in doped OSCs^42^. Importantly, the doping kinetics are nearly identical with or without TFSI^−^ (Fig. 1e and Extended Data Fig. 3b), demonstrating that counterion exchange is not the rate-limiting step. Instead, the doping rate is strongly dependent on OSC film thickness, with thicker films requiring longer times to reach saturation conductivity and fully bleach the neutral absorption peak (Extended Data Fig. 5 and Supplementary Fig. 8), indicating that GAP doping is probably limited by the diffusion of persulfate throughout the bulk of the OSC layer. Overall, these results indicate that TFSI^−^ acts as a stabilizing counterion that facilitates ion exchange and thereby substantially enhances the overall efficiency of the GAP doping process without altering the doping kinetics.

**Extended Data Fig. 6:**
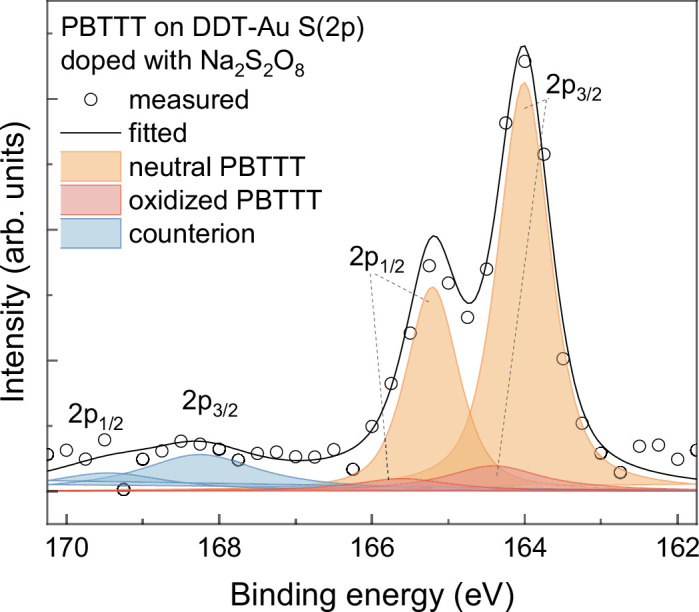
Doping level of PBTTT on DDT-modified gold. XPS S(2p) spectrum of PBTTT deposited on DDT-treated gold and doped with Na2S2O8/LiTFSI. The doping level is estimated to be 12% based on the ratio of the oxidized PBTTT peak area to the total PBTTT S(2p) signal. Source data

To investigate the role of gold in promoting the doping of PBTTT by persulfate, we conducted X-ray photoelectron spectroscopy (XPS) measurements. As shown in Fig. 1f, the sulfur S(2*p*) signal of undoped PBTTT spin-cast on a gold-coated glass substrate exhibits two symmetrical peaks at 164.0 eV and 165.2 eV, corresponding to the spin-split doublet (2*p*_3/2_ and 2*p*_1/2_) of the sulfur atom in the thiophene ring. After exposure to Na_2_S_2_O_8_/LiTFSI solution, the S(2*p*) peaks of PBTTT shift to lower binding energy and display an asymmetric tail extending towards higher binding energy. This asymmetry suggests the formation of positive charges (polarons/bipolarons) delocalized along the PBTTT backbone, reflecting changes in the electronic environment near the sulfur atom^43,44^. In addition, a strong S(2*p*) signal attributed to counterions appears at 167–171 eV, along with distinct nitrogen N(1*s*) and fluorine F(1*s*) signals (Supplementary Fig. 9). By contrast, when PBTTT films were exposed to Na_2_S_2_O_8_/LiTFSI solution without an underlying gold layer, the S(2*p*) peaks remained largely unchanged and the TFSI^−^ signal was weak, indicating a substantially lower doping level. To quantify the doping level, the PBTTT S(2*p*) region was fitted with two doublets corresponding to neutral and oxidized polythiophene^16^. The estimated doping levels were 45% with gold, 11% without gold and 12% with DDT-modified gold (see Extended Data Fig. 6 for the latter), calculated as the ratio of the oxidized PBTTT doublet area to the total area of both oxidized and neutral components. The increase in charge density correlates with the higher conductivity and lower Seebeck coefficient measured for PBTTT films doped on bare gold electrodes (Fig. 1e and Supplementary Fig. 10). For comparison, PBTTT films doped via F_4_TCNQ/ion exchange exhibited a doping level of 33% (Supplementary Fig. 11), consistent with their lower conductivity (Supplementary Fig. 4). These trends are further supported by electron paramagnetic resonance (EPR) measurements, which showed a strong signal only when gold was present (Fig. 1g), and by ultraviolet photoelectron spectroscopy (UPS) measurements, where PBTTT films deposited on gold exhibited a greater shift in the secondary electron cut-off and a higher work function (5.25 eV versus 4.66 eV; Fig. 1h). In addition, the near alignment of the valence band edge with the Fermi level confirmed a heavily doped state in PBTTT films on gold (Supplementary Fig. 12).

Grazing-incidence wide-angle X-ray scattering (GIWAXS) provided further structural evidence. Both pristine and doped PBTTT films exhibited an edge-on orientation on gold-coated and bare silicon substrates (Fig. 1i). However, only the doped films on gold showed notable structural changes: the π–π stacking peak shifted from *q*_*xy*_ = 1.697 to 1.742 Å^−1^ (corresponding to a reduced stacking distance from 3.70 to 3.61 Å), and the lamellar peak shifted from *q*_*z*_ = 0.781 to 0.748 Å^−1^ (increased spacing from 24.14 to 25.20 Å), consistent with the incorporation of TFSI^−^ counterions in the side-chain region^13,15^. By contrast, films doped on bare silicon exhibited only minimal structural changes, indicating weak oxidation in the absence of gold (Supplementary Figs. 13 and 14 and Supplementary Table 1). Taken together, the XPS, EPR, UPS and GIWAXS results provide compelling evidence that the presence of gold during doping enables markedly more efficient oxidation of PBTTT with persulfate.

## Mechanism of GAP doping

**Fig. 2:**
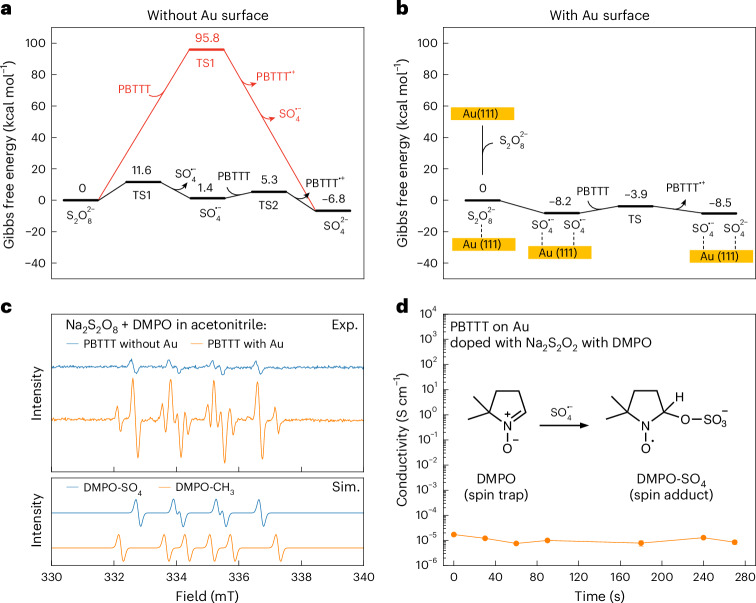
Mechanism of GAP doping process. **a**,**b**, DFT-calculated Gibbs free energy profiles for the reaction of persulfate with PBTTT in the absence (**a**) and presence (**b**) of a gold surface. In **a**, both a direct electron-transfer pathway (red line) and a two-step radical-mediated mechanism (black line) were considered. **c**, EPR spectra of Na_2_S_2_O_8_ solution in contact with a PBTTT film using DMPO as a spin trap, confirming the presence of ${\rm{SO}}_4^{\cdot-}$ radicals. Simulated (Sim.) spectra are shown for comparison and exhibit good agreement with the experimental (Exp.) data. **d**, Electrical conductivity of PBTTT films doped with Na_2_S_2_O_8_ in the presence of DMPO, showing suppression of doping due to radical scavenging. The chemical structures of DMPO and its adduct with ${\rm{SO}}_4^{\cdot-}$ are reported in **d**. Points represent mean values; error bars show standard deviation (s.d., not visible at this scale); *n* = 10 independent samples. Source data

To gain mechanistic insight into the gold-assisted doping of PBTTT by Na_2_S_2_O_8_, we performed DFT calculations to identify the most energetically favourable charge-transfer pathway, both with and without a gold surface. In the absence of gold, two possible doping mechanisms were considered (Fig. 2a). The first involves direct electron transfer between S_2_O_8_^2−^ and PBTTT (red curve), resulting in the formation of a radical species (S_2_O_8_^•3−^), which spontaneously dissociates into SO_4_^2−^ and SO_4_^•−^. The second is a two-step process (black curve), where S_2_O_8_^2−^ undergoes homolytic cleavage to yield two SO_4_^•−^ radicals, followed by electron transfer between one of the radicals and the PBTTT chain. The energy profiles indicate that the direct pathway is less favourable, with a substantially higher activation barrier than the two-step route. These results align with previous reports^45^, highlighting the ease with which symmetric S_2_O_8_^2−^ dissociates into radical species in solution.

Given this preference, we next modelled the two-step mechanism in the presence of a gold surface (Fig. 2b). Remarkably, adsorption of S_2_O_8_^2−^ onto gold substantially lowers the energy barriers for both homolytic cleavage and subsequent electron transfer, with the cleavage step becoming entirely barrierless. This catalytic effect is probably due to gold’s ability to stabilize the radical intermediates and delocalize the excess charge resulting from the oxidation of PBTTT. Thus, the presence of gold not only facilitates the generation of reactive sulfate radicals but also lowers the overall energetic cost of the doping process. These theoretical findings support our experimental observations and confirm the critical role of gold as a catalyst in the activation of persulfate dopants. In the absence of gold, doping is inefficient; in its presence, radical generation and electron transfer proceed readily. Although the fate of the sulfate species after electron transfer has not been investigated, our results provide compelling evidence for a gold-assisted radical-doping mechanism.

The presence of SO_4_^•−^ radicals was confirmed by EPR spectroscopy using 5,5-dimethyl-1-pyrroline-*N*-oxide (DMPO), a spin trap that forms stable nitroxide adducts with radical species. As shown in Fig. 2c, the EPR spectrum of Na_2_S_2_O_8_ solution in the presence of PBTTT film on gold exhibits four prominent peaks with an intensity ratio of 1:1:1:1 and hyperfine coupling constants of *α*_N_ = 1.35 mT and *α*_H_ = 1.20 mT. These values are consistent with those reported for the DMPO-SO_4_ adduct^46,47^, confirming the generation of SO_4_^•−^ radicals. In addition, six weaker peaks of equal intensity were observed, characterized by hyperfine coupling constants of *α*_N_ = 1.46 mT and *α*_H_ = 2.12 mT. These values correspond to the DMPO-CH_3_ adduct^48^, probably resulting from acetonitrile degradation induced by the highly reactive SO_4_^•−^ radicals^49^. The experimental spectra closely match the simulated results (Fig. 2c). Notably, the EPR spectra of the same solution in the presence of PBTTT but without gold showed similar peak patterns but with markedly lower intensity, indicating a reduced concentration of SO_4_^•−^ radicals formed under those conditions. To confirm the critical role of SO_4_^•−^ radicals in the doping process, PBTTT films were immersed in a Na_2_S_2_O_8_ solution containing DMPO, and their electrical conductivity was measured. As shown in Fig. 2d, no change in conductivity was observed, which can be attributed to the scavenging of SO_4_^•−^ radicals by DMPO, thereby inhibiting the doping of PBTTT.

## Generality of the GAP doping process

**Fig. 3:**
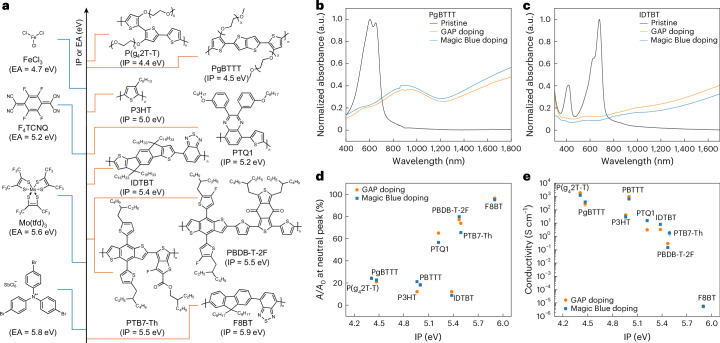
Generality of the GAP doping process. **a**, Chemical structures and IP of representative p-type semiconducting polymers, along with the EA of commonly used molecular p-dopants. **b**,**c**, Absorption spectra of PgBTTT (**b**) and IDTBT (**c**) polymers doped with Na_2_S_2_O_8_ and Magic Blue, showing comparable spectral responses. **d**, Ratio of the neutral peak absorbance before and after doping with Na_2_S_2_O_8_ and Magic Blue, indicating pronounced bleaching for polymers with IP <5.2 eV and much less pronounced bleaching for those with IP >5.2 eV. **e**, Electrical conductivity as a function of IP for the polymers shown in **a**, doped with Na_2_S_2_O_8_ and Magic Blue. Points, mean; error bars, s.d. (not visible); *n* = 10 independent samples. Source data

Next, we evaluated the generality of the GAP doping process across a range of semiconducting polymers with IPs spanning approximately 1.5 eV, as measured by cyclic voltammetry (Supplementary Fig. 15), and featuring either hydrophilic or hydrophobic side chains. The set included poly(3,3′-bis(tetraethylene glycol methyl)-2,2′-dithiophene-thiophene) (P(g_4_2T-T)), PgBTTT (a glycolated derivative of PBTTT), poly(3-hexylthiophene) (P3HT), poly(indacenodithiophene-co-benzothiadiazole) (IDTBT), poly[2,3-bis(3-octyloxyphenyl)-5,8-quinoxalinediyl-2,5-thiophenediyl] (PTQ1), poly[4,8-bis(5-(2-ethylhexyl)thiophen-2-yl)benzo[1,2-b;4,5-b′]dithiophene-2,6-diyl-alt-(4-(2-ethylhexyl)-3-fluorothieno[3,4-b]thiophene-)-2-carboxylate-2-6-diyl)] (PTB7-Th), poly[(2,6-(4,8-bis(5-(2-ethylhexyl-3-fluoro)thiophen-2-yl)-benzo[1,2-b:4,5-b′]dithiophene))-alt-(5,5-(1′,3′-di-2-thienyl-5′,7′-bis(2-ethylhexyl)benzo[1′,2′-c:4′,5′-c′]dithiophene-4,8-dione)] (PBDB-T-2F) and poly(9,9-dioctylfluorene-alt-benzothiadiazole) (F8BT) (Fig. 3a). For comparison, the electron affinities (EAs) of conventional molecular p-dopants such as FeCl_3_, F_4_TCNQ, Mo(tfd)_3_ and tris(4-bromophenyl)ammoniumyl hexachloroantimonate (Magic Blue) are also reported in Fig. 3a. Notably, GAP doping induced optical changes in the absorption spectra that closely resembled those caused by the strong oxidant Magic Blue (EA ~5.8 eV)^50^ across all polymers tested (Fig. 3b–d), including the high-IP polymer PBDB-T-2F (IP ~5.5 eV) (Supplementary Figs. 16 and 17).

**Extended Data Fig. 7:**
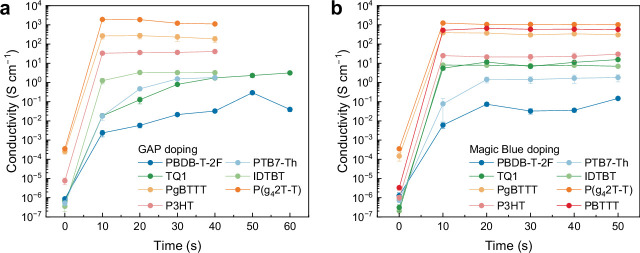
Electrical conductivity of GAP-doped polymers and comparison with Magic Blue. Electrical conductivity of representative p-type polymers doped with (**a**) GAP and (**b**) Magic Blue. Points represent mean values; error bars show standard deviation (s.d., not visible at this scale); n = 10 independent samples. Source data

While the spectral changes were less pronounced for higher-IP polymers such as F8BT (IP ~5.9 eV; Supplementary Fig. 18), the effect on conductivity was still evident. For example, doping F8BT with persulfate resulted in a 50–70-fold increase in conductivity, comparable to that achieved with Magic Blue. More broadly, the conductivity of persulfate-doped polymers decreased systematically with increasing IP, from 1,908 ± 114 S cm^−1^ for P(g_4_2T-T) (IP ~4.4 eV) to 0.29 ± 0.05 S cm^−1^ for PBDB-T-2F (Fig. 3e). For the latter, this corresponds to a conductivity enhancement of nearly five orders of magnitude compared with its pristine state (Extended Data Fig. 7 and Supplementary Table 2). These findings indicate that the sulfate radical acts as a potent oxidant, at least on par with Magic Blue or Mo(tfd)_3_ (EA ~5.6 eV)^51^, although its standard reduction potential (*E*° (SO_4_^•−^/SO_4_^2−^) = 2.4 V versus normal hydrogen electrode)^52^ suggests that its oxidizing power may, in fact, be even greater.

## Lateral doping gradient and applicability of the GAP doping process

**Extended Data Fig. 8:**
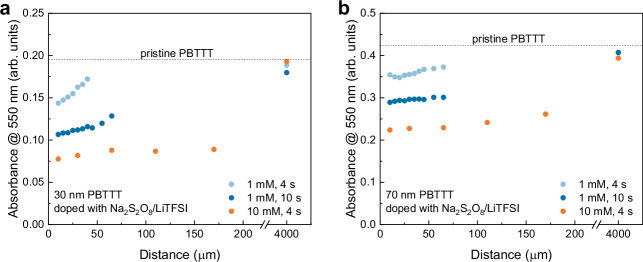
Spatial doping gradient in PBTTT films. Evolution of the neutral PBTTT absorption peak at 550 nm as a function of distance from the gold edge for (**a**) thin (~30 nm) and (**b**) thick (~70 nm) films. The data were extracted from Supplementary Fig. 20. Source data

GAP doping initiates at the gold–OSC interface and propagates laterally, forming a continuous doping gradient across the OSC film. This behaviour is visible macroscopically (Supplementary Video 2) and confirmed by Kelvin probe mapping over millimetre-scale regions under high-concentration (10 mM), long-time doping conditions (up to 10 min; Supplementary Fig. 19). To resolve the gradient at smaller length scales, we performed spatially resolved visible-range absorbance mapping near the gold edge with a 5-µm step (Extended Data Fig. 8 and Supplementary Fig. 20). At high dopant concentrations (10 mM) and short immersion times (4 s), SO_4_^•−^ induces rapid bleaching, fully doping the film up to ~170 μm from the electrode edge, while regions 4 mm away remain undoped. By contrast, lowering the dopant concentration to 1 mM produces a much more confined lateral diffusion: under these mild conditions, a well-defined ~40-μm doping gradient in PBTTT is observed after 4 s. Extending the doping time to 10 s broadens the gradient further, consistent with a laterally advancing doping front. Thicker OSC films exhibit more gradual gradients due to slower persulfate penetration, in agreement with the electrical data showing that lower dopant concentrations or greater film thickness require longer times to reach maximum conductivity (Extended Data Fig. 2 and Supplementary Fig. 8). Raman mapping further corroborates this behaviour (Supplementary Fig. 21). Together, these results demonstrate that GAP doping enables tunable lateral doping fronts ranging from tens of micrometres to several millimetres, depending on dopant concentration, treatment time and film thickness.

**Fig. 4:**
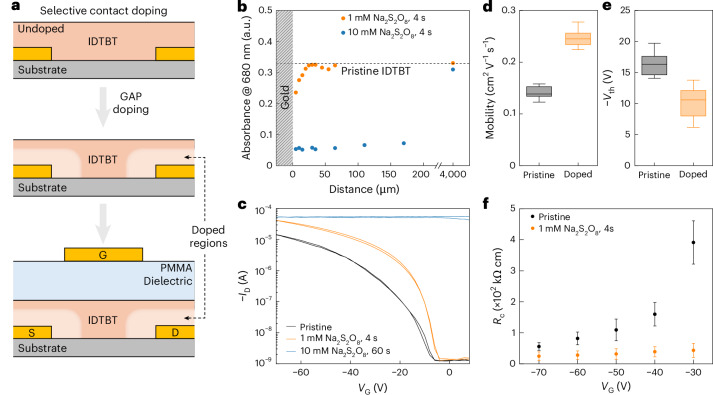
Applicability of the GAP doping in OFETs. **a**, Schematic illustration of selective contact doping in OFETs enabled by GAP. **b**, Evolution of the IDTBT absorption peak at 680 nm as a function of distance from the gold electrode edge. **c**, Transfer curves of pristine and contact-doped IDTBT-based OFETs. **d**,**e**, Mobility (**d**) and threshold voltage (**e**) of pristine and contact-doped IDTBT-based OFETs. The contact-doped devices were treated with 1 mM Na_2_S_2_O_8_ for 4 s. The data were collected from devices with a channel length *L* = 50 μm and a width *W* = 2 mm at *V*_DS_ = −70 V. Boxes, median line and cap indicate 25/75 percentile, mean and minimum/maximum values, respectively; *n* = 6 independent samples. **f**, Contact resistance as a function of gate voltage before and after contact doping. Points represent mean values; error bars show standard deviation; *n* = 4 independent samples. Source data

**Extended Data Fig. 9:**
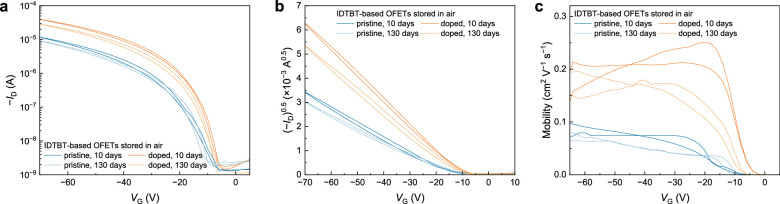
Stability of GAP-doped IDTBT-based OFETs. (**a**) Transfer, (**b**) *I*_D_^0.5^, and (**c**) saturation mobility curves of pristine and doped IDTBT OFETs (*L* = 50 μm, *W* = 2 mm, *V*_DS_ = − 70 V) monitored for up to 130 days of storage under ambient laboratory conditions (≈22 °C, ∼40% RH). Doping conditions: 1 mM Na_2_S_2_O_8_ for 4 s. Source data

Inspired by this distinctive behaviour, we applied GAP doping to OFETs to achieve controlled, contact-proximal doping of the source/drain regions (Fig. 4a). By using a low concentration of Na_2_S_2_O_8_ (1 mM) and a short immersion time (4 s), we formed lateral doping gradients in IDTBT, confined to within ~20 μm of the electrode edge (Fig. 4b and Supplementary Fig. 22). This selective contact doping resulted in a twofold increase in on-current, while the off-current remained nearly unchanged^53^ (Fig. 4c). By contrast, applying a higher concentration of Na_2_S_2_O_8_ (10 mM) and longer doping time (60 s) led to complete channel doping and loss of transistor behaviour. This partial doping near the contacts improved overall device performance by mitigating nonideal OFET behaviour, with the field-effect hole mobility increasing from 0.14 ± 0.02 cm^2^ V^−1^ s^−1^ to 0.25 ± 0.04 cm^2^ V^−1^ s^−1^ and the threshold voltage shifting from −17.36 ± 2.87 V to −9.87 ± 3.78 V (Fig. 4d,e, Supplementary Fig. 23 and Supplementary Table 3). The devices remained stable in ambient conditions for at least 10 days without noticeable degradation and retained more than 60% of their initial drain current after 130 days of storage in air (Extended Data Fig. 9). Contact resistance, extracted via the transfer length method (Fig. 4f and Supplementary Fig. 24), revealed that undoped devices exhibit high contact resistance at low gate bias (391.25 ± 69.71 kΩ cm at *V*_G_ = − 30 V), which decreased with increasing gate voltage. By contrast, GAP-doped devices showed substantially lower contact resistance even at low gate voltage (43.29 ± 22.62 kΩ cm at *V*_G_ = − 30 V). Corresponding output characteristics are shown in Supplementary Fig. 25. Notably, this gradual contact-proximal doping is difficult to achieve with conventional solution-based dopants such as Magic Blue, which tend to uniformly dope the entire channel (Supplementary Fig. 26).

## Outlook

In summary, we developed a GAP doping mechanism in which clean gold surfaces promote the homolytic cleavage of persulfate to generate SO_4_^•−^ radicals, as supported by DFT calculations and EPR measurements. These radicals efficiently p-dope a wide range of OSCs with IPs spanning over 1.5 eV, achieving conductivities approaching 1,900 S cm^−1^. The localized activation of persulfate at the gold–OSC interface induces a lateral doping gradient, confirmed by spectroscopic analysis, enabling selective doping of source and drain regions in OFETs, reducing contact resistance and enhancing mobility. Notably, the spatial resolution of the GAP doping method, on the order of a few tens of micrometres under mild conditions, is comparable to evaporation- or printing-based approaches (Extended Data Table 1), while offering the advantages of simplicity, full solution compatibility and mask-free processing. Importantly, the use of persulfate, an inexpensive and readily available oxidant, makes the GAP doping method scalable, residue-free and cost-effective, providing a practical route for large-area and scalable organic electronics.

## Methods

### Materials

PBTTT^54^ and its glycolated derivative PgBTTT^55^ were synthesized according to previous reports. P3HT, LiTFSI, persulfate salts, DDT, DMPO, Magic Blue, F_4_TCNQ, poly(methyl methacrylate) (PMMA) and organic solvents such as acetonitrile, 1,2-dichlorobenzene, chloroform and ethanol were purchased from Sigma-Aldrich. P(g_4_2T-T), IDTBT, PTB7-Th and PBDB-T-2F were purchased from 1-Material. PTQ1 was purchased from Solarmer Energy, while F8BT was purchased from Ossila.

### Sample preparation

PBTTT was dissolved in 1,2-dichlorobenzene (10 mg ml^−1^) and spin-coated onto the substrates at 2,000 rpm. The films were annealed at 180 °C for 20 min in a N_2_-filled glovebox and then slowly cooled to room temperature. Under these conditions, PBTTT films had a typical thickness of ~30 nm. Thicker films were obtained from a more concentrated solution (20 mg ml^−1^), with spin-coating at 1,000 rpm yielding ~100 nm and at 2,000 rpm yielding ~70 nm. All other p-type polymers were dissolved in either 1,2-dichlorobenzene or chloroform (10 mg ml^−1^) and spin-coated following the same procedure used for PBTTT.

### Doping process

Na_2_S_2_O_8_ was mixed with LiTFSI in acetonitrile at a molar ratio of 1:10. Typically, a 10-mM dopant solution was prepared by dissolving Na_2_S_2_O_8_ (4.76 mg, 0.02 mmol) and LiTFSI (57.4 mg, 0.2 mmol) in 2 ml of acetonitrile. The resulting dopant solution was sonicated at room temperature for 20 min. A freshly prepared dopant solution was used for each doping experiment. The polymer films were immersed in the dopant solution, rinsed with deionized water, and subsequently dried under N_2_. Doping with Magic Blue and F_4_TCNQ was performed following previously reported procedures^42,50^.

### Electrical characterization

Electrical conductivity measurements were performed using a four-probe set-up with a channel width/length of 5 mm/0.5 mm. The results were validated using devices with longer channels (5 mm/1 mm and 5 mm/2 mm; Supplementary Fig. 27). Seebeck coefficient measurements were performed in a glovebox using a pair of Peltier elements to generate the temperature difference. All measurements were conducted using a Keithley 4200-SCS semiconductor characterization system.

For the gold electrode, 5-nm Cr and 50-nm Au were thermally evaporated onto a cleaned glass substrate and patterned using photolithography. The DDT-modified gold was prepared by immersing bare gold electrodes in a DDT solution (0.25 vol% in ethanol) for 3 days, followed by ethanol washing. DDT modification was confirmed by contact angle measurements (Supplementary Fig. 28). Aluminium, silver, chromium and copper electrodes (50 nm) were thermally evaporated onto glass substrates, using a 5-nm Cr adhesion layer, and subsequently patterned by photolithography. The ITO electrode, approximately 150 nm thick, was purchased from Zhuhai Kaivo Optoelectronic Technology and patterned by laser etching. The sheet resistance of Au and ITO electrodes was about 1 and 7 Ω sq^−1^, respectively.

### Optical characterization

PBTTT films were prepared by spin-coating the polymer solution on glass substrates with or without gold strips. Gold was thermally evaporated onto cleaned glass and patterned by photolithography, with a 500-μm spacing between adjacent lines. The absorbance spectra were recorded using a ultraviolet–visible–near-infrared spectrophotometer (PerkinElmer Lambda 950) with a 2-nm step. Spatially resolved absorbance mapping was performed in transmission mode using a Nikon Eclipse L200N (10× objective) coupled to a fibre-optic spectrophotometer (50-µm fibre, QE PRO-ABS, Ocean Optics). The microscope’s built-in halogen lamp served as the light source. The recorded spectra were referenced against an uncoated glass substrate. All other p-type polymer films were deposited and characterized following the same procedure used for PBTTT.

### XPS and UPS spectroscopy

XPS and UPS measurements were performed on PBTTT films spin-coated onto ITO- or gold-coated glass substrates using a Scienta ESCA 200 system equipped with a SES 200 electron analyser under ultrahigh vacuum conditions (1 × 10^−10^ mbar). A monochromatic Al Ka X-ray source (1,486.6 eV) and a helium discharge lamp (21.22 eV) were used for XPS and UPS measurements, respectively. All spectra were collected at normal emission and calibrated by a sputter-cleaned Au film, with the Fermi level set to 0 eV and the Au 4*f* peak at 84.0 eV.

### EPR measurements

The EPR spectra were acquired using a SPINSCAN X spectrometer by Linev Systems in the dark at room temperature. The modulation frequency, microwave power and microwave frequency were set to 100 kHz, 1 mW and 9.46 GHz, respectively. For polaron measurements in doped PBTTT films, the PBTTT solution was spin-coated onto poly(ethylene terephthalate) (PET) or gold-coated PET substrates. Then, the PBTTT films were cut into pieces measuring 2 × 20 mm and placed into an N_2_-filled quartz tube. The spectra were recorded with a modulation width of 0.7 mT and a sweep time of 60 s. To test for SO_4_^•−^ radicals in solution, 100 μl of DMPO was added to the persulfate solution. After 5 min, the mixtures were transferred into capillaries, which were sealed at the bottom and inserted into an N_2_-filled quartz tube. EPR spectra were recorded with a modulation width of 0.1 mT and a sweep time of 300 s. The EPR signals of the DMPO adducts were simulated using the open-source software package EasySpin (version 6.0.10).

### GIWAXS measurements

GIWAXS measurements were performed on PBTTT films spin-coated onto silicon or gold-coated silicon substrates. The diffraction patterns were collected at an X-ray energy of 11.06 eV with an incidence angle of 0.12° at Beamline 9A in the Pohang Accelerator Laboratory. All samples were measured under vacuum with an exposure time of 10 s.

### Kelvin probe

Work function mapping was performed using a scanning Kelvin Probe (SKP5050) under ambient conditions. The measurement was calibrated using highly ordered pyrolytic graphite (work function 4.6 eV), with a scanning step of 0.4 mm.

### QCM-D measurements

Quartz crystals coated with gold (QSX301, Biolin Scientific) or SiO_2_ (QSX303, Biolin Scientific) were used as substrates for PBTTT deposition. The frequency and dissipation of the QCM crystals were recorded using a QSense Analyzer (Biolin Scientific). The pristine crystal served as a reference to the reported frequency and dissipation shifts, which were recorded following PBTTT coating and subsequent contact with the dopant solution (10 mM Na_2_S_2_O_8_ in acetonitrile).

### Cyclic voltammetry

Cyclic voltammetry was performed using a BioLogic SP-200 potentiostat. A 0.1-M solution of Bu_4_NPF_6_ in acetonitrile was used as electrolyte. Polymer films were spin-coated onto clean gold substrates, which served as the working electrode. A platinum mesh and an Ag/AgCl (saturated) electrode were used as the counter and reference electrodes. The potential was calibrated using a standard ferrocene sample. All scans were recorded at a scan rate of 50 mV s^−1^.

### Raman spectroscopy

Raman spectra were collected at room temperature using a Renishaw InVia Reflex confocal Raman spectrometer equipped with a Leica 50× objective (numerical aperture 0.50), a 2,400 grooves mm^−1^ grating and an Ar-ion 532-nm laser (100 mW at the source). A Renishaw charge-coupled device camera was used as the detector. Spectra were acquired in static mode, covering the spectral range 583–1,760 cm^−1^. The exposure time was 1 s, with one accumulation for pristine samples and four accumulations for doped samples. This configuration provides a spectral resolution better than 1 cm^−1^ and a spatial resolution of ~1.3 μm. The spectrometer was calibrated to the first-order Si vibrational mode at 520.6 cm^−1^. For Raman mapping, a step size of 2 μm was used, with mapped regions including both on- and off-electrode areas. For each point, the intensity ratio *P*_1_/*P*_2_ (Supplementary Fig. 21) is reported after applying a constant subtraction to set the signal to zero at 1,228 cm^−1^.

### Computational details

DFT-based simulations were performed with the CP2K/Quickstep package, using a hybrid Gaussian and plane wave method^56^. A triple quality TZVP Gaussian basis set was used for the Au and Na atoms, and a triple quality aug-TZVP Gaussian basis set augmented with a diffusive function was used for all other atoms^57^. The Goedecker–Teter–Hutter pseudopotentials^58^, together with a 400-Ry plane wave cut-off, were used to expand the densities obtained with the Perdew–Burke–Ernzerhof^59^ exchange-correlation density functional, and van der Waals forces are taken into account with the Grimme D3 Method^60^. Only the gamma point was considered in a supercell approach. Periodic boundary conditions are applied in all directions of space.

#### Surface model

The gold (*111*) surface was constructed using a slab model with three Au layers, yielding a 17.31 Å × 14.99 Å surface unit cell (108 Au atoms). A 35-Å vacuum region between the slabs was used to minimize unrealistic interactions between the slabs. Only the gamma point was considered in a supercell approach. Periodic boundary conditions were applied in all directions of space. The slab charge was neutralized, including two Na cations to balance the S_2_O_8_^2−^ negative charge. The transition state of the homolytic cleavage of S_2_O_8_^2−^ was identified by tracking the energy as a function of the O–O peroxo bond distance, while the transition state of the electron transfer was calculated by considering the optimized geometries of the reactants with the electronic configuration of the products. Molecular graphics were produced by the CHEMCRAFT graphical package^61^.

#### Gibbs free energy profile

Potential energy as obtained by the self-consistent field (SCF) procedure at zero Kelvin is adopted as energy reference. Entropic and enthalpic contributions were evaluated on the analysed reactions without the gold surface by performing the frequency calculation of the molecular species at 298.15 K and 1 atm as implemented in the G16 code^62^. G16 calculations were performed at the M06 hybrid functional level^63^, using the standard all-electron 6–311+G** basis set for all atoms. The enthalpic and entropic contributions were then ‘appended’ to the SCF energy profile of the analogue reactions that occurred on the gold surface to obtain the Gibbs free energy profile. Solvation contribution to the energy profile was evaluated within the PCM approach^64^ for molecular species using acetonitrile (*ε* = 38.8 D) as a prototypical solvent. For the reaction on the gold surface, the solvent effect was taken into account by considering the solvation energy values associated with half-cavity formation.

#### OFET fabrication and characterization

Top-gate, bottom-contact OFETs were fabricated on glass substrates with photolithographically defined Cr/Au electrode (5/50 nm thickness), as schematically illustrated in Supplementary Fig. 29. IDTBT was then deposited by spin-coating from 7 mg ml^−1^ chlorobenzene solution at 1,500 rpm for 40 s, followed by thermal annealing at 100 °C for 30 min to remove residual solvent. The contact doping was performed using the above-described method. Subsequently, a PMMA dielectric layer (*M*_W_ = 120 kDa, 80 mg ml^−1^ in butylacetate) was spin-coated onto the IDTBT layer at 3,000 rpm for 40 s, followed by drying on a hotplate at 80 °C for 2 h. A 40-nm-thick aluminium top gate was then thermally evaporated through a shadow mask to complete the device. All transistor characteristics were measured in air using a Keithley 4200-SCS semiconductor characterization system. A summary of methods used to spatially control doping of OSCs is provided in Extended Data Table 1 (refs. ^26,28–35,65–68^).

## Online content

Any methods, additional references, Nature Portfolio reporting summaries, source data, extended data, supplementary information, acknowledgements, peer review information; details of author contributions and competing interests; and statements of data and code availability are available at 10.1038/s41563-026-02547-0.

## Supplementary information

Supplementary Information Supplementary Figs. 1–29, Tables 1–3 and References.

Supplementary Video 1 Video showing that rapid Na_2_S_2_O_8_ doping occurs in the presence of Au electrodes.

Supplementary Video 2 Video demonstrating a macroscopic lateral doping gradient across the film.

## Source data

Source Data Fig. 1 Source data for Fig. 1.

Source Data Fig. 2 Source data for Fig. 2.

Source Data Fig. 3 Source data for Fig. 3.

Source Data Fig. 4 Source data for Fig. 4.

Source Data Extended Data Fig. 1 Source data for Extended Data Fig. 1.

Source Data Extended Data Fig. 2 Source data for Extended Data Fig. 2.

Source Data Extended Data Fig. 3 Source data for Extended Data Fig. 3.

Source Data Extended Data Fig. 5 Source data for Extended Data Fig. 5.

Source Data Extended Data Fig. 6 Source data for Extended Data Fig. 6.

Source Data Extended Data Fig. 7 Source data for Extended Data Fig. 7.

Source Data Extended Data Fig. 8 Source data for Extended Data Fig. 8.

Source Data Extended Data Fig. 9 Source data for Extended Data Fig. 9.

## Figures and Tables

**Extended Data Table 1:**
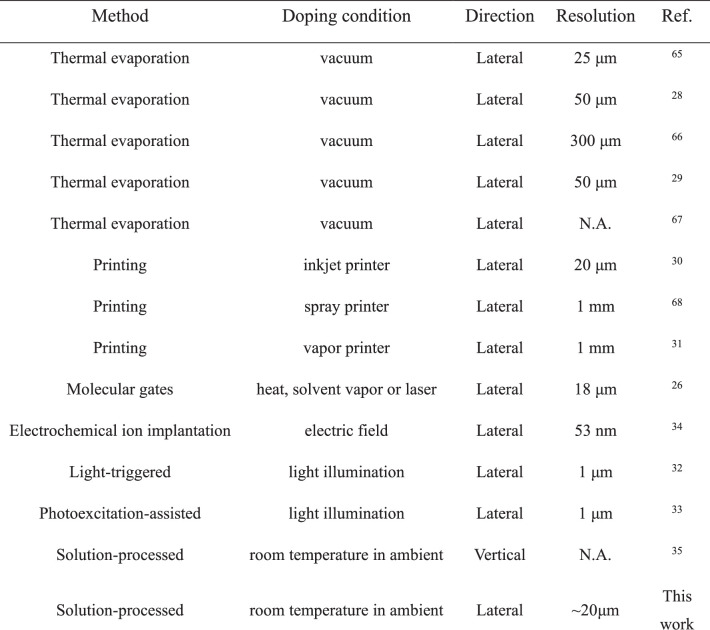
Summary of methods for spatially controlled doping of OSCs

## Data Availability

The data supporting the findings of this study are available within the Article and its Supplementary Information. Source data are provided with this paper.
