# Supplementary material for: Gold-activated persulfate p-doping of organic semiconductors
Source: Nat Mater. 2026 Mar 17;25(8):1429–36. doi: 10.1038/s41563-026-02547-0 (PMC13421340; doi:10.1038/s41563-026-02547-0)
Supplement: Supplementary file 1 — Supplementary Figs. 1–29, Tables 1–3 and References. [file 41563_2026_2547_MOESM1_ESM.pdf]

nature materials

Supplementary information

https://doi.org/10.1038/s41563-026-02547-0

Gold-activated persulfate p-doping of organic semiconductors

In the format provided by the authors and unedited

Content

Supplementary Fig. 1	Photographs of pristine and doped PBTTT films.
Supplementary Fig. 2	QCM-D measurements of PBTTT films.
Supplementary Fig. 3	Persulfate doping of PBTTT with different metals.
Supplementary Fig. 4	Conductivity of F_4TCNQ-doped PBTTT.
Supplementary Fig. 5	Conductivity of $Na_2S_2O_8$-doped PBTTT (ITO electrodes).
Supplementary Fig. 6	PBTTT doping with various persulfate salts.
Supplementary Fig. 7	Effect of LiTFSI on PBTTT.
Supplementary Fig. 8	Thickness dependence of GAP-doped PBTTT with/without TFSI.
Supplementary Fig. 9	XPS analysis of GAP-doped PBTTT.
Supplementary Fig. 10	Seebeck coefficient of GAP-doped PBTTT.
Supplementary Fig. 11	XPS analysis of F_4TCNQ-doped PBTTT.
Supplementary Fig. 12	UPS of GAP-doped PBTTT.
Supplementary Fig. 13	GIWAXS of GAP-doped PBTTT.
Supplementary Fig. 14	GIWAXS analysis of GAP-doped PBTTT.
Supplementary Fig. 15	Ionization potentials of various p-type OSCs measured by CV.
Supplementary Fig. 16	Absorption spectra of various GAP-doped p-OSCs.
Supplementary Fig. 17	Absorption spectra of various p-OSCs doped by Magic Blue.
Supplementary Fig. 18	Comparison of GAP and Magic Blue doping in F8BT.
Supplementary Fig. 19	Macroscopic lateral doping gradient in GAP-doped PBTTT.
Supplementary Fig. 20	Absorbance mapping of GAP-doped PBTTT.
Supplementary Fig. 21	Raman microscopy of GAP-doped PBTTT.
Supplementary Fig. 22	Absorbance mapping of GAP-doped IDTBT.
Supplementary Fig. 23	Electrical characteristics of GAP-doped IDTBT OFETs.
Supplementary Fig. 24	Transfer length method for IDTBT OFETs.
Supplementary Fig. 25	Output characteristics of IDTBT OFETs.
Supplementary Fig. 26	Transfer characteristics of IDTBT OFETs doped with Magic Blue.
Supplementary Fig. 27	Channel-length dependence of PBTTT conductivity.
Supplementary Fig. 28	Contact angle measurements.
Supplementary Fig. 29	Optical micrograph of the OFET.
Supplementary Table 1	Summary of the GIWAXS analysis.
Supplementary Table 2	Summary of conductivity for the GAP-doped p-type OSCs.
Supplementary Table 3	Key performance metrics of IDTBT OFETs.
Supplementary References	

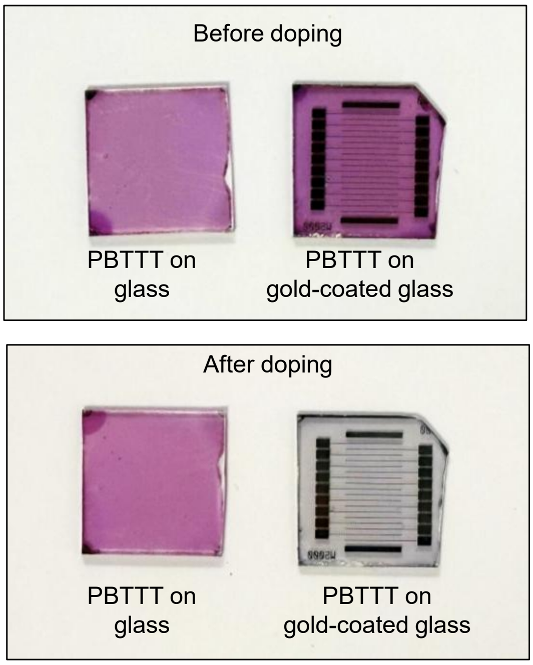

Supplementary Fig. 1 | Photographs of pristine and doped PBTTT films. Pristine PBTTT films show a similar purple-red color on both glass and gold-coated glass. After immersion in a $Na_2S_2O_8$/LiTFSI solution (10/100 mM in acetonitrile) for 1 min, the PBTTT film on gold-coated glass becomes fully bleached, whereas the film on plain glass retains its original color.

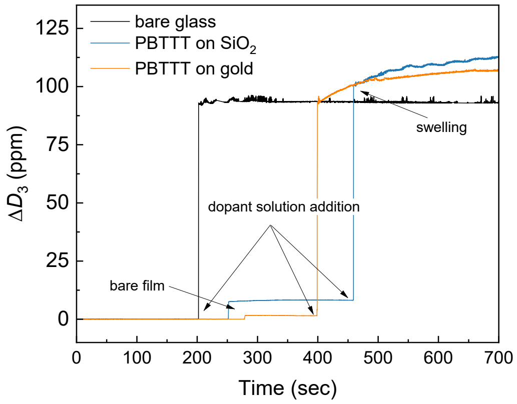

Supplementary Fig. 2 | QCM-D measurements of PBTTT films. Dissipation changes for the 3rd overtone measured by QCM-D for different stages: from the clean crystal, to the crystal with a bare PBTTT film, and finally with the dopant solution applied on top. Data are shown for both glass and gold-coated surfaces.

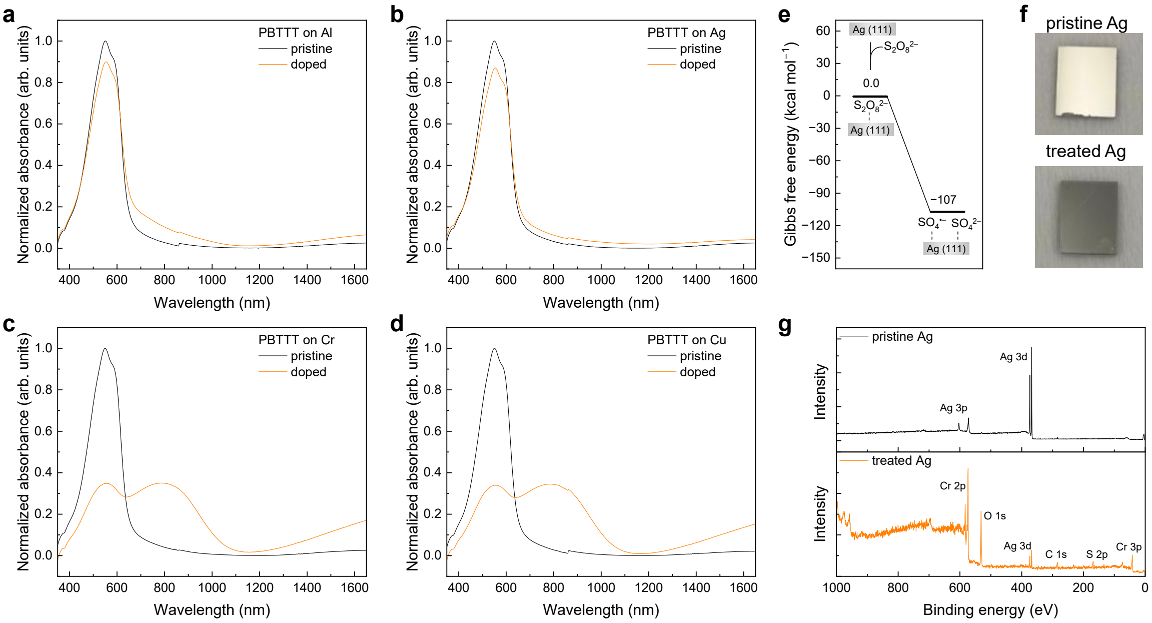

Supplementary Fig. 3 | Persulfate doping of PBTTT with different metals. (a-d) UV-vis-NIR absorption spectra of PBTTT films (~30 nm) deposited on four metals, (a) aluminum, (b) silver, (c) chromium, and (d) copper, and doped with 10 mM $Na_2S_2O_8$ in the presence of LiTFSI for 1 min. Strong doping signatures (bleaching of the neutral peak at 550 nm and emergence of a broad polaron band >1100 nm) are observed for Cr and Cu, whereas Al and Ag show only weak responses. All metals (50 nm) were thermally evaporated on glass substrates with an additional 5 nm chromium adhesion layer. (e) DFT-calculated Gibbs free-energy profile for persulfate activation by PBTTT in the presence of a silver surface. (f) Optical images of pristine and persulfate-treated Ag films, indicating surface oxidation and leaching. (g) XPS survey spectra of Ag before and after exposure to 10 mM $Na_2S_2O_8$ in the presence of LiTFSI for 1 min, corroborating the observed surface changes.

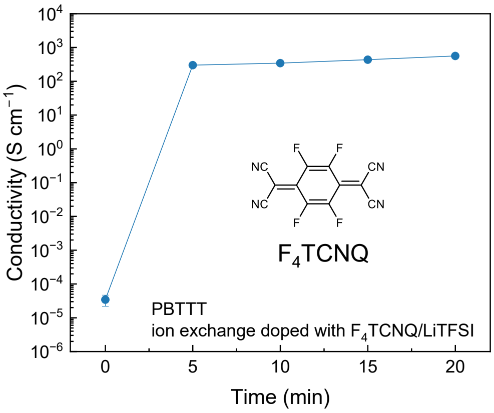

Supplementary Fig. 4 | Conductivity of F_4TCNQ-doped PBTTT. Electrical conductivity of PBTTT films doped with F_4TCNQ in the presence of LiTFSI, using the ion-exchange doping method. The chemical structure of F_4TCNQ is shown in the inset. Points represent mean values; error bars show standard deviation; n = 10 independent samples.

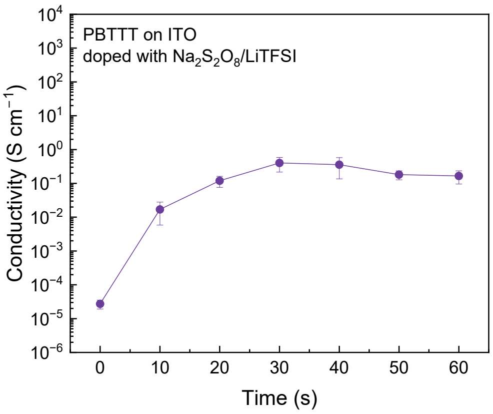

Supplementary Fig. 5 | Conductivity of $Na_2S_2O_8$-doped PBTTT (ITO electrodes). Electrical conductivity of PBTTT films doped with $Na_2S_2O_8$ in the presence of LiTFSI, measured using ITO electrodes. Points represent mean values; error bars show standard deviation; n = 10 independent samples.

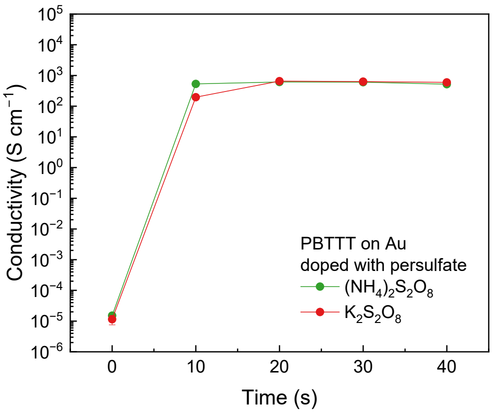

Supplementary Fig. 6 | PBTTT doping with various persulfate salts. Electrical conductivity of PBTTT films doped with $K_2S_2O_8$ or $(NH_4)_2S_2O_8$ in the presence of LiTFSI, measured using gold electrodes. Points represent mean values; error bars show standard deviation (s.d., not visible at this scale); n = 10 independent samples.

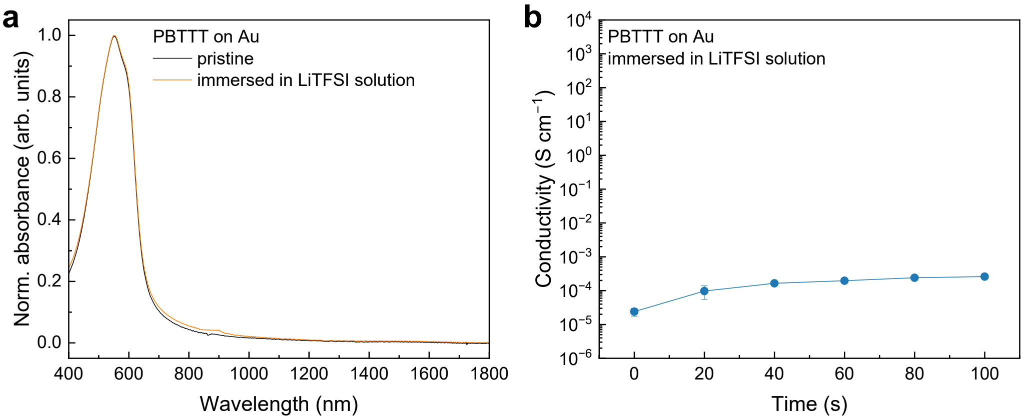

Supplementary Fig. 7 | Effect of LiTFSI on PBTTT. (**a**) Absorption spectra and (**b**) electrical conductivity of PBTTT films after immersion in a LiTFSI solution (0.1 M in acetonitrile) in the presence of gold. LiTFSI alone does not induce detectable doping. Points represent mean values; error bars show standard deviation (s.d., not visible at this scale); n = 10 independent samples.

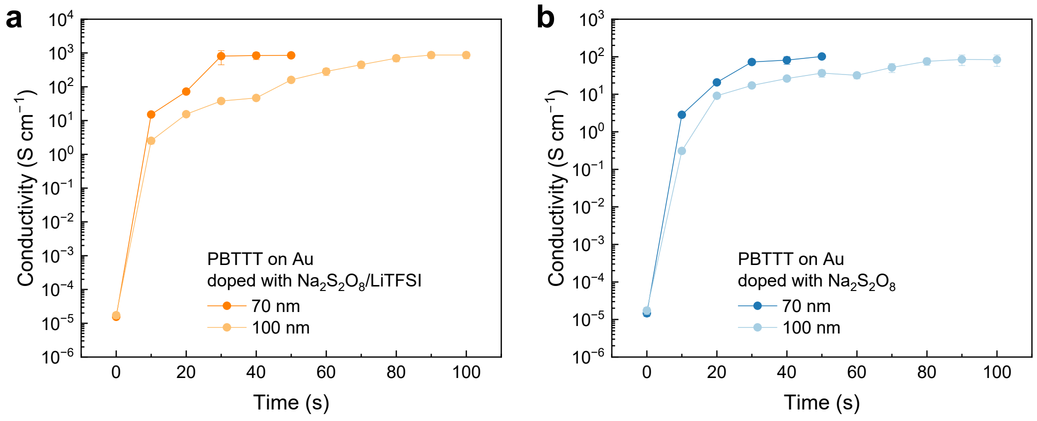

Supplementary Fig. 8 | Thickness dependence of GAP-doped PBTTT with/without TFSI. Electrical conductivity of PBTTT films doped with $Na_2S_2O_8$ with (**a**) and without (**b**) LiTFSI, measured using Au electrodes. Points represent mean values; error bars show standard deviation (s.d., not visible at this scale); n = 10 independent samples.

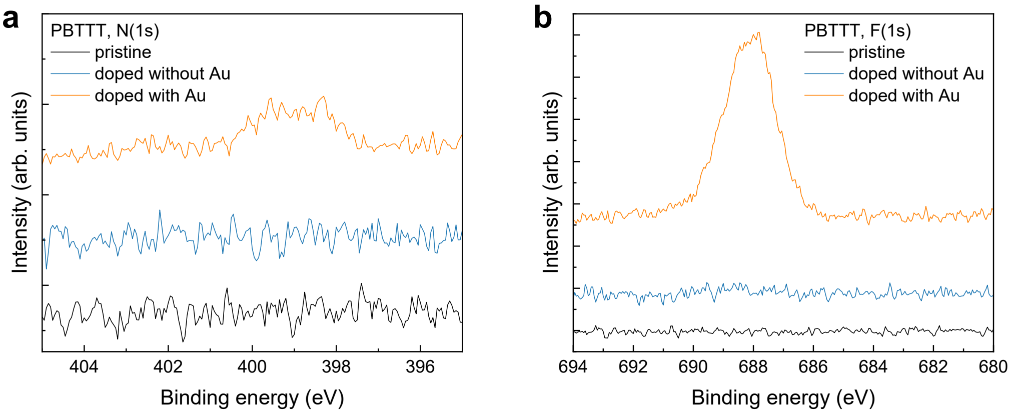

Supplementary Fig. 9 | XPS analysis of GAP-doped PBTTT. (**a**) N(1s) and (**b**) F(1s) signals in pristine PBTTT and GAP-doped PBTTT in the presence and absence of gold. The results show the presence of $TFSI^-$ anions, which act as counterions to balance the positively charged PBTTT backbone.

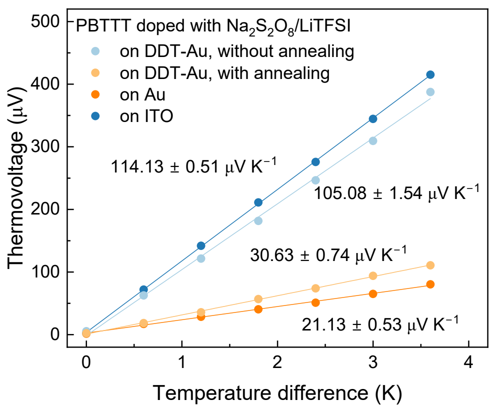

Supplementary Fig. 10 | Seebeck coefficient of GAP-doped PBTTT. Thermovoltage versus temperature difference for PBTTT doped with $Na_2S_2O_8$ in the presence of LiTFSI, measured using gold, DDT-modified gold, and ITO electrodes. For DDT-modified gold, both annealed and non-annealed samples were measured.

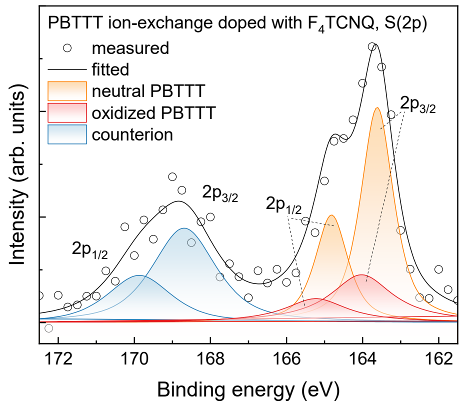

Supplementary Fig. 11 | XPS analysis of F_4TCNQ-doped PBTTT. XPS S(2p) spectrum of PBTTT doped via conventional F_4TCNQ ion-exchange method using LiTFSI as the counterion. The doping level is estimated to be 32% based on the ratio of the oxidized PBTTT peak area to the total PBTTT S(2p) signal.

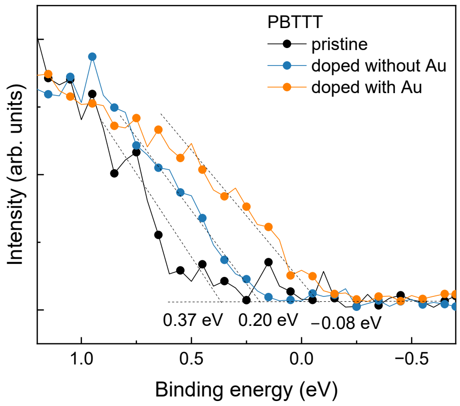

Supplementary Fig. 12 | UPS of GAP-doped PBTTT. UPS spectra of pristine and persulfate-doped PBTTT films, with and without underlying gold, highlighting the low binding energy region to illustrate changes in the electronic structure upon doping.

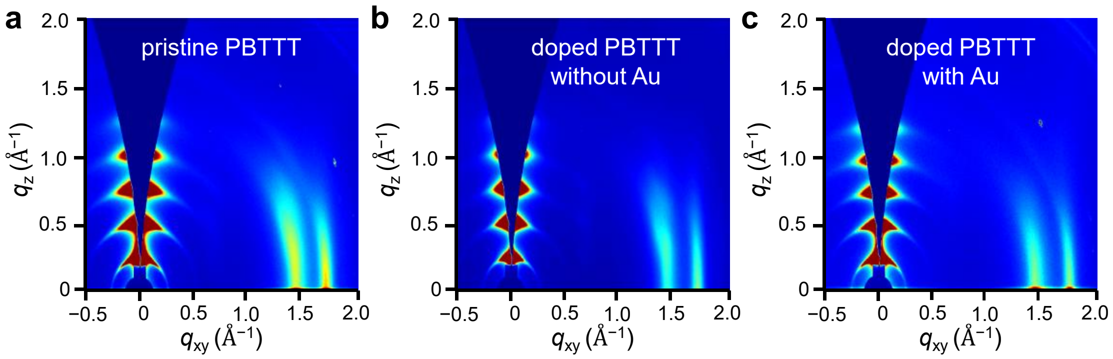

Supplementary Fig. 13 | GIWAXS of GAP-doped PBTTT. 2D GIWAXS scattering images of (**a**) pristine PBTTT, (**b**) persulfate-doped PBTTT without underlying gold, and (**c**) persulfate-doped PBTTT on gold.

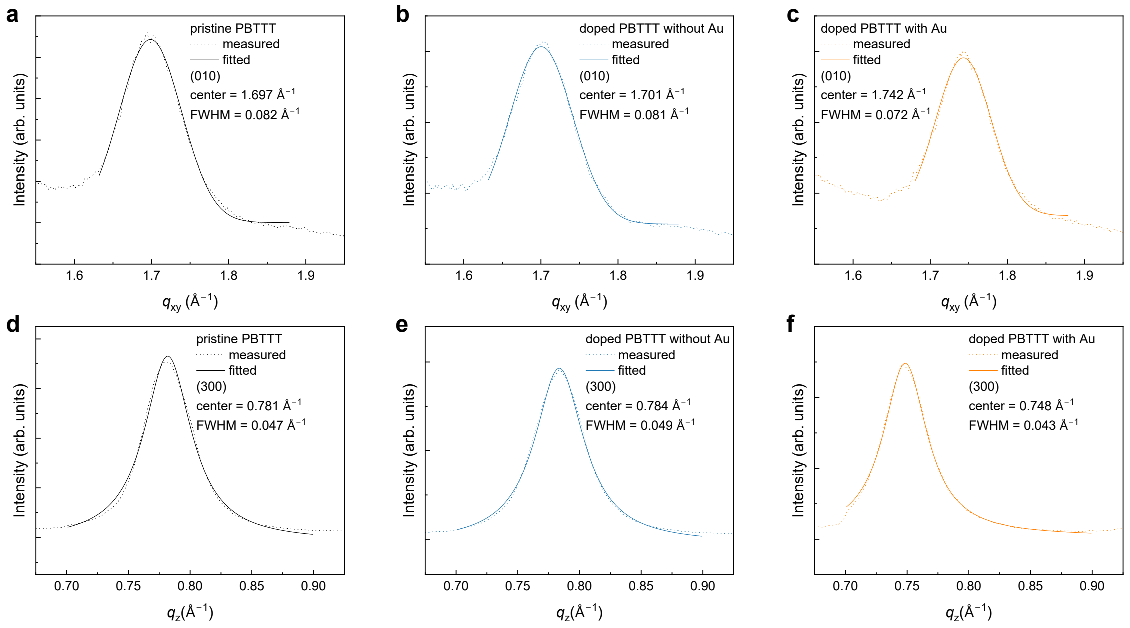

Supplementary Fig. 14 | GIWAXS analysis of GAP-doped PBTTT. π-π stacking diffraction peaks for (**a**) pristine PBTTT and persulfate-doped PBTTT films (**c**) without and (**e**) with gold. Lamellar packing diffraction peaks for (**b**) pristine PBTTT and persulfate-doped PBTTT films (**d**) without and (**f**) with gold.

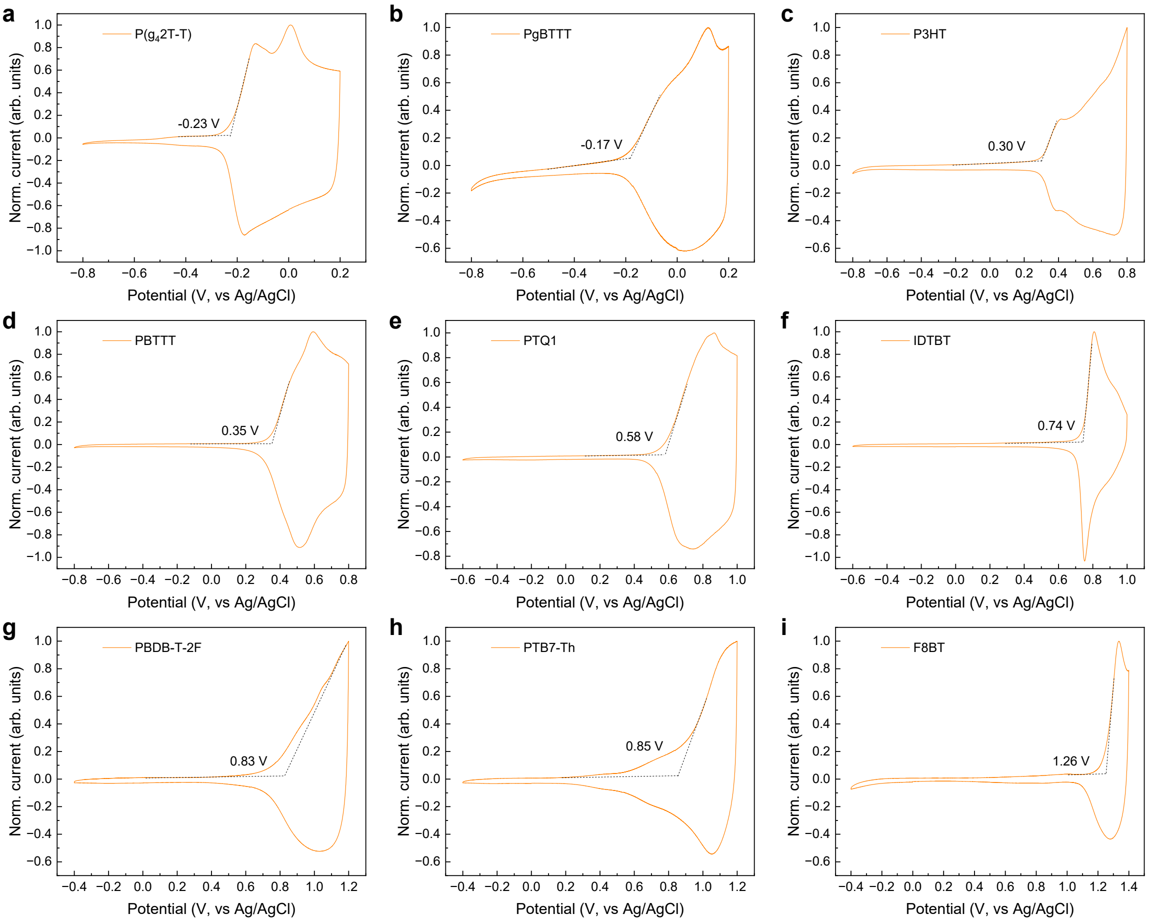

Supplementary Fig. 15 | Ionization potentials of various p-type OSCs measured by CV. Cyclic voltammograms of (**a**) P(g$_4$2T-T), (**b**) PgBTTT, (**c**) P3HT, (**d**) PBTTT, (**e**) PTQ1, (**f**) IDTBT, (**g**) PBDB-T-2F, (**h**) PTB7-Th, and (**i**) F8BT films measured in 0.1 M Bu_4NPF_6 in acetonitrile. Scan rate: 50 mV s^{-1}.

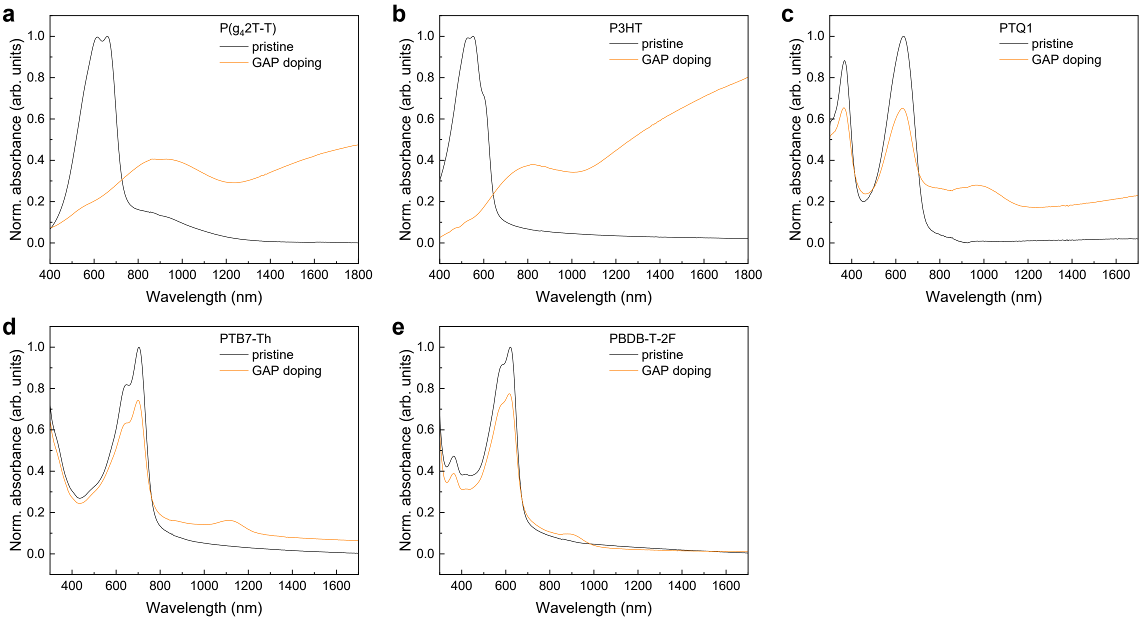

Supplementary Fig. 16 | Absorption spectra of various GAP-doped p-OSCs. UV-Vis-NIR absorption spectra of (**a**) P($g_4$2T-T), (**b**) P3HT, (**c**) PTQ1, (**d**) PTB7-Th, and (**e**) PBDB-T-2F.

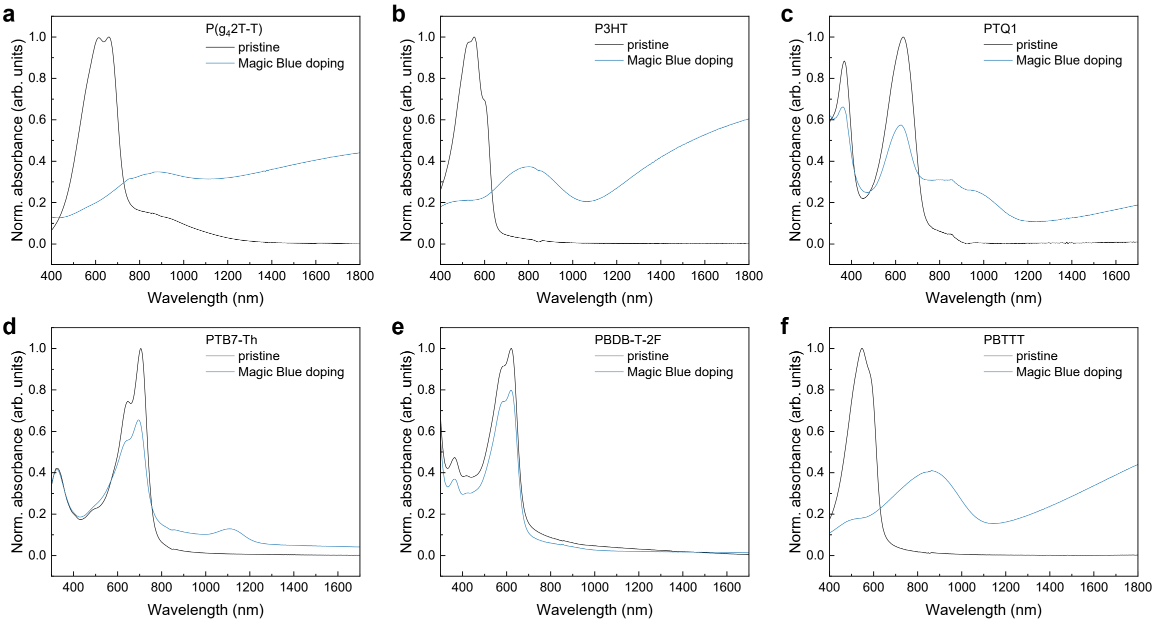

Supplementary Fig. 17 | Absorption spectra of various p-OSCs doped by Magic Blue. UV-Vis-NIR absorption spectra of (**a**) P($g_4$2T-T), (**b**) P3HT, (**c**) PTQ1, (**d**) PTB7-Th, (**e**) PBDB-T-2F, and (**f**) PBTTT.

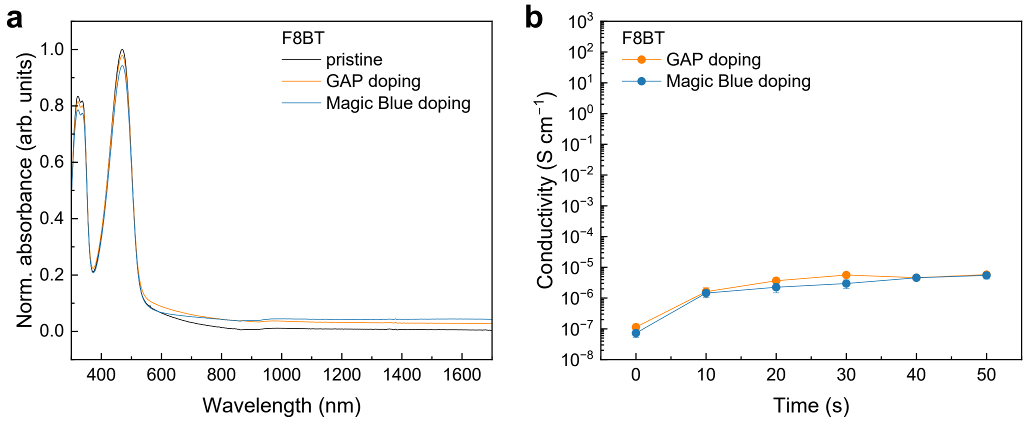

Supplementary Fig. 18 | Comparison of GAP and Magic Blue doping in F8BT. (**a**) UV-vis-NIR absorption spectra and (**b**) electrical conductivity of F8BT films deposited on gold-coated glass and doped with either persulfate or Magic Blue. Points represent mean values; error bars show standard deviation (s.d., not visible at this scale); n = 10 independent samples.

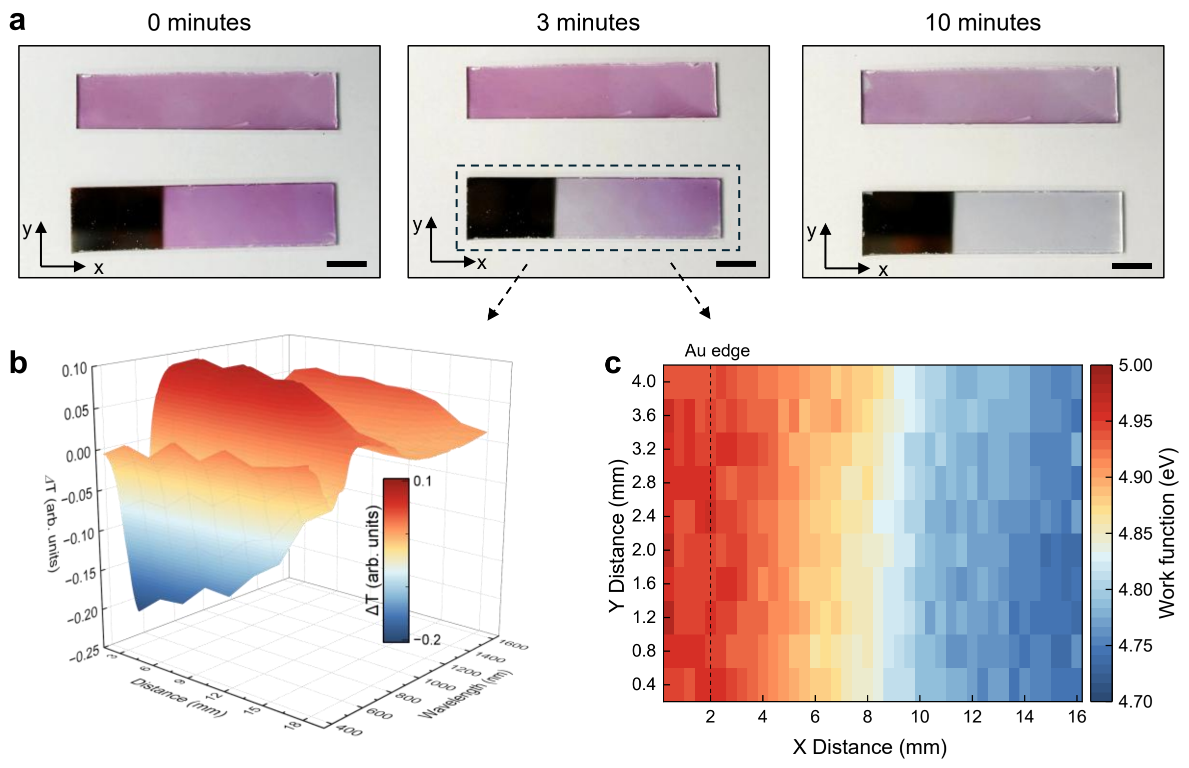

Supplementary Fig. 19 | Macroscopic lateral doping gradient in GAP-doped PBTTT. (a) Photographs of PBTTT films on glass and half gold-coated substrates after 3 min immersion in persulfate solution (scale bar: 0.5 mm). (**b**) Three-dimensional map of differential transmission as a function of wavelength and distance from the gold edge, showing the spatial evolution of doping. (**c**) Work function mapping of the PBTTT film on the half-coated substrate. Doping initiates at the gold/PBTTT interface, where the film becomes more transparent, while regions farther from the gold edge retain their red-purple color. The gradual increase in transparency and elevated work function near the gold confirms the formation of a lateral doping gradient. After 10 min, the entire film becomes bleached, indicating complete doping.

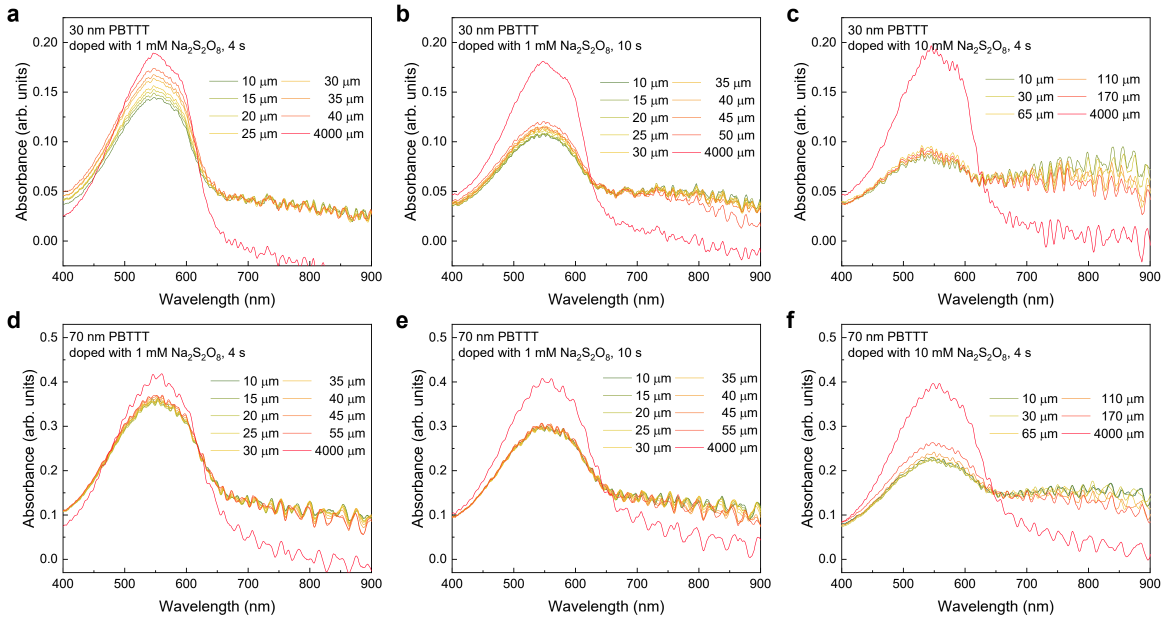

Supplementary Fig. 20 | Absorbance mapping of GAP-doped PBTTT. UV-Vis absorption spectra of (**a-c**) thin PBTTT films (30 nm) doped with (**a**) 1 mM $Na_2S_2O_8$ for 4 s, (**b**) 1 mM $Na_2S_2O_8$ for 10 s, and (**c**) 10 mM $Na_2S_2O_8$ for 4 s, and (**d-f**) thick PBTTT film (70 nm) doped with (**d**) 1 mM $Na_2S_2O_8$ for 4 s, (**e**) 1 mM $Na_2S_2O_8$ for 10 s, and (**f**) 10 mM $Na_2S_2O_8$ for 4 s. All doping experiments were performed in the presence of LiTFSI.

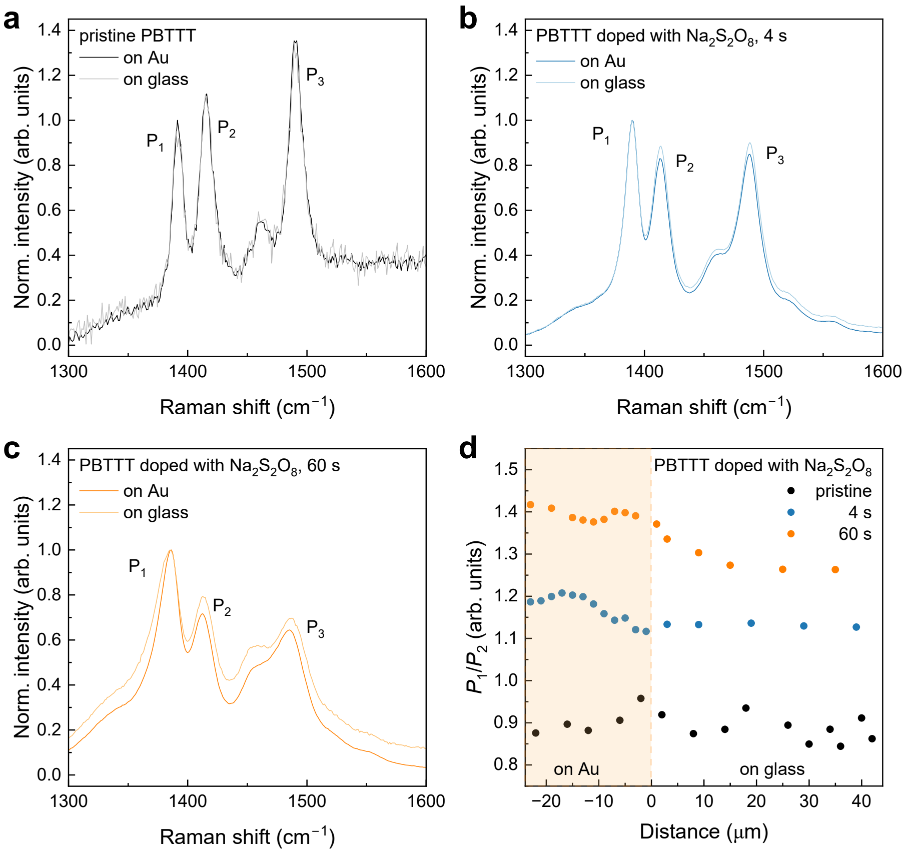

Supplementary Fig. 21 | Raman microscopy of GAP-doped PBTTT. Raman spectra of (**a**) pristine PBTTT films and PBTTT films doped with 10 mM $Na_2S_2O_8$/LiTFSI for (**b**) 4 s and (**c**) 60 s on gold or glass substrate. All samples exhibit three characteristic peaks: P_1 at ~1389 cm^{-1}, P_2 at 1413 cm^{-1}, and P_3 at 1488 cm^{-1}. Peaks P_1 and P_2 have strong contributions from the C=C stretching modes in the thienothiophene/thiophene units, whereas peak P_3 arises predominantly from the C=C/C−C stretching/shrinking mode[1]. All spectra are shown after normalization to the P_1 peak intensity. For pristine PBTTT, the Raman spectra are identical on gold and glass. After doping, the P_1/P_2 ratio increases, consistent with previous reports on chemical/electrochemical doping of PBTTT[1,2]. Moreover, the P_1/P_2 ratio is consistently higher on gold, indicating a higher doping level. (**d**) Spatial evolution of the P_1/P_2 ratio as a function of distance from the gold edge. In pristine PBTTT, the P_1/P_2 ratio remains nearly constant across the scanned region. After 4 s of doping, a clear gradient in the P_1/P_2 ratio appears near the gold edge. Increasing the doping time further increases the P_1/P_2 ratio and extends the propagation of the doping front.

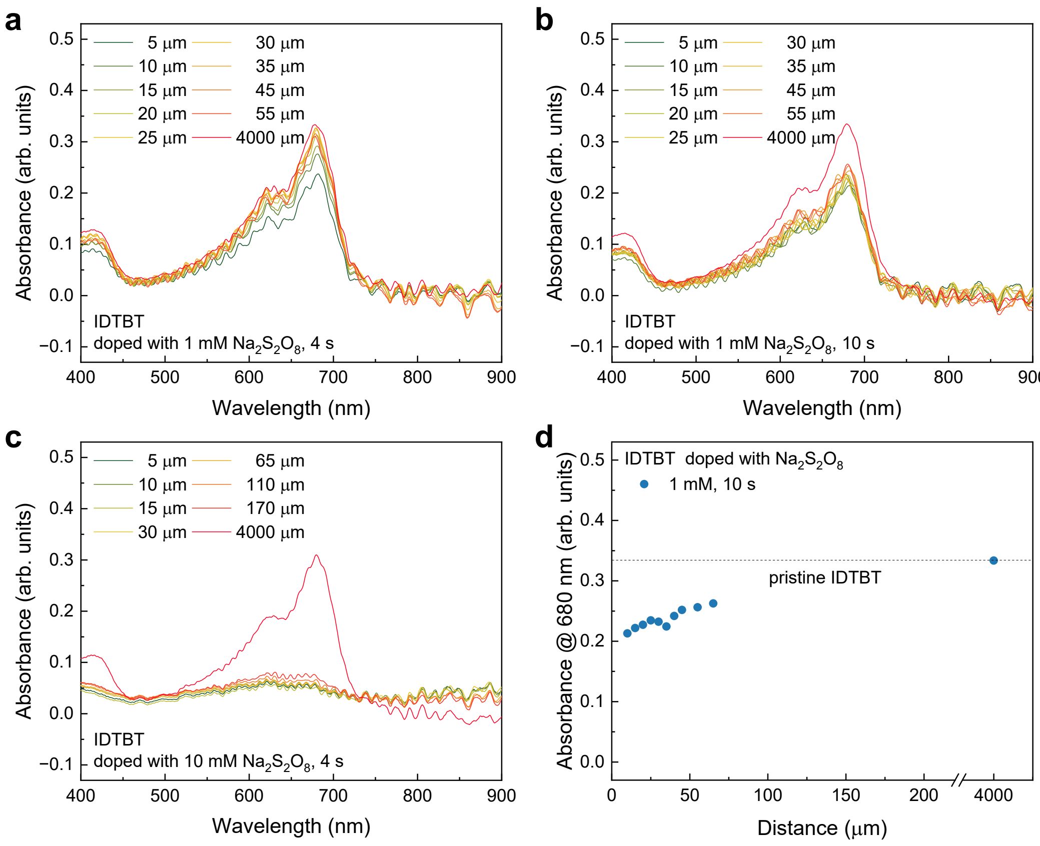

Supplementary Fig. 22 | Absorbance mapping of GAP-doped IDTBT. UV-Vis absorption spectra of IDTBT doped with (**a**) 1 mM $Na_2S_2O_8$/LiTFSI for 4 s, (**b**) 1 mM $Na_2S_2O_8$ for 10 s, and (**c**) 10 mM $Na_2S_2O_8$ for 4 s. (**d**) Evolution of the IDTBT absorption peak at 680 nm as a function of distance from the gold edge.

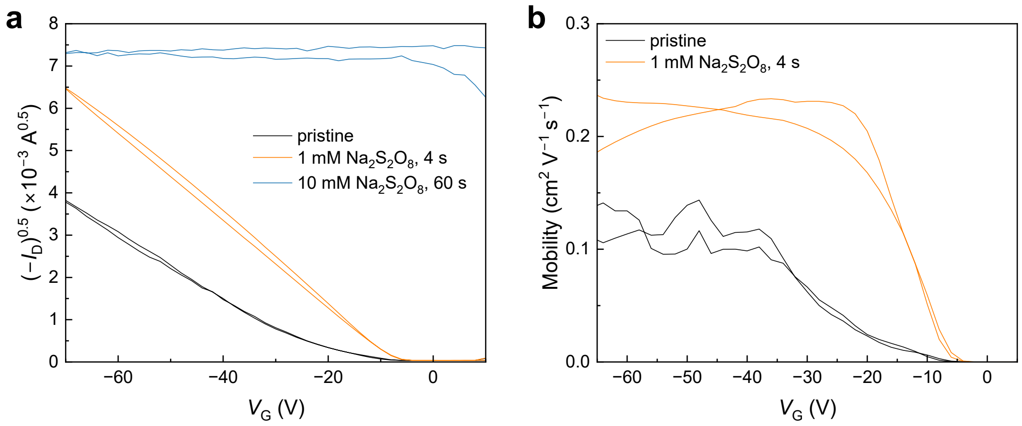

Supplementary Fig. 23 | Electrical characteristics of GAP-doped IDTBT OFETs. (**a**) $I_D^{0.5}$ and (**b**) extracted saturation mobility of the pristine and GAP-doped IDTBT OFETs shown in Fig. 4c.

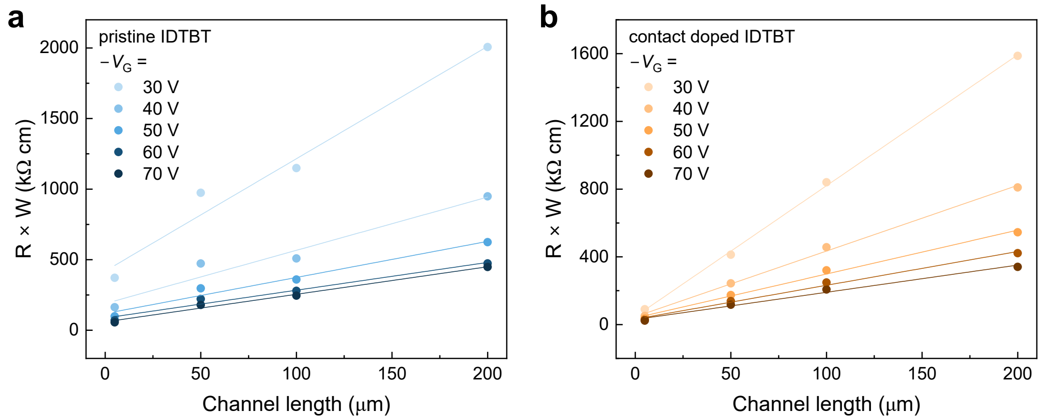

Supplementary Fig. 24 | Transfer length method for IDTBT OFETs. Width-normalized total resistance of OFET devices based on (**a**) pristine IDTBT and (**b**) IDTBT with selectively GAP-doped contact regions, measured at various channel lengths at V_D = –10 V.

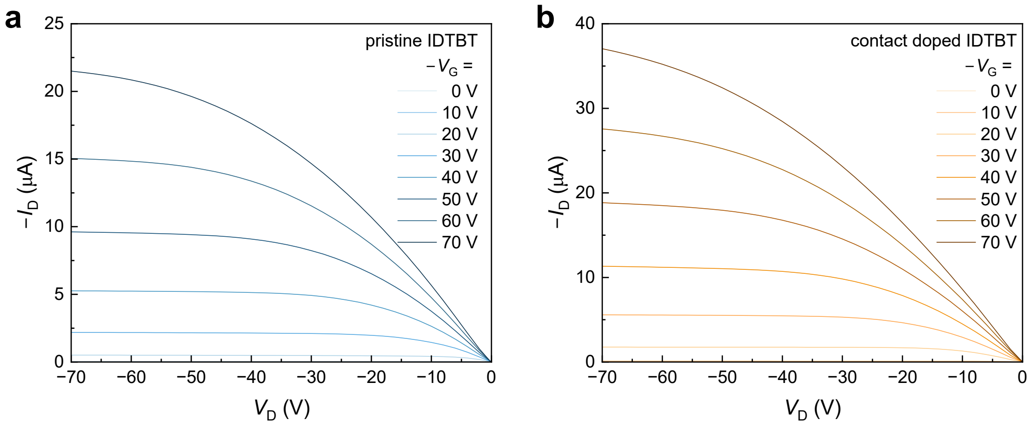

Supplementary Fig. 25 | Output characteristics of IDTBT OFETs. Output curves of pristine (**a**) and GAP contact-doped (**b**) IDTBT OFETs (L = 50 μm, W = 2 mm). The increased I_D upon contact doping confirms enhanced on-state performance.

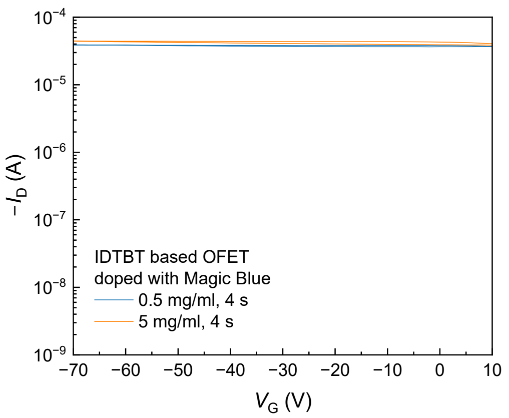

Supplementary Fig. 26 | Transfer characteristics of IDTBT OFETs doped with Magic Blue. Even at a low concentration (0.5 mg mL^{-1}) and short exposure time (4 s), IDTBT is fully doped due to the strong oxidizing nature of Magic Blue and its inability to selectively dope only the contact regions.

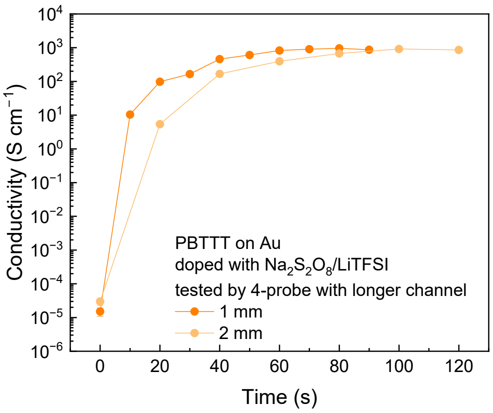

Supplementary Fig. 27 | Channel-length dependence of PBTTT conductivity. Four-probe conductivity measurements of PBTTT doped with $Na_2S_2O_8$ in the presence of LiTFSI with L = 1 mm or 2 mm (W = 5 mm). Longer channels require longer doping times to reach maximum conductivity, while the final values are nearly identical to those for shorter-channel devices (L = 0.5 mm; Fig. 1e). Points represent mean values; error bars show standard deviation (s.d., not visible at this scale); n = 10 independent samples.

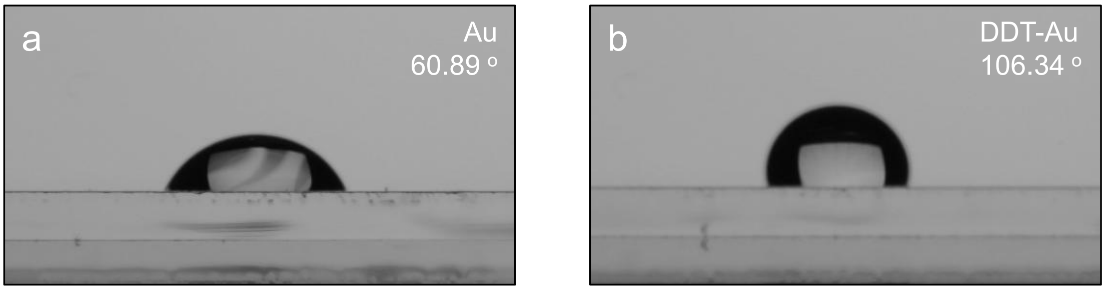

Supplementary Fig. 28 | Contact angle measurements. Water droplets on (**a**) bare gold and (**b**) gold modified with a self-assembled monolayer of DDT, demonstrating increased surface hydrophobicity after modification.

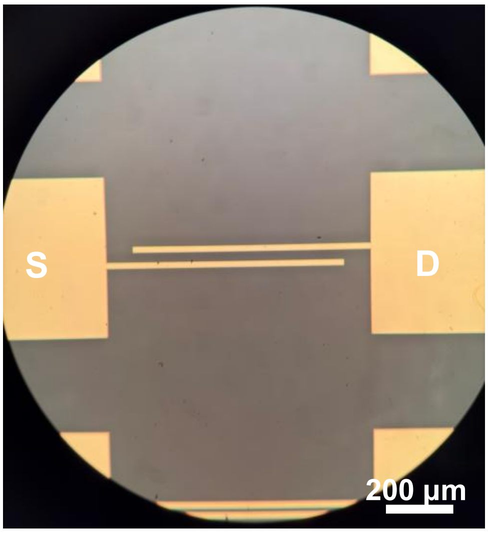

Supplementary Fig. 29 | Optical micrograph of the OFET. Top-view optical micrograph of the OFET substrate used in this study.

Supplementary Table 1 | Summary of the GIWAXS analysis. Calculated packing distances, coherence lengths, and paracrystalline disorder parameters for lamellar and π-π stacking of PBTTT films.

	π-π stacking				Lamellar stacking			
	q_{xy} (010) (Å^{-1})	Distance (Å)	Coherence length (Å)	Paracrystalline disorder	q_z (300) (Å^{-1})	Distance (Å)	Coherence length (Å)	Paracrystalline disorder
Undoped PBTTT	1.697	3.703	68.961	0.0877	0.781	24.135	120.316	0.0979
Doped PBTTT without Au	1.701	3.693	69.813	0.0871	0.784	24.042	115.405	0.0997
Doped PBTTT with Au	1.742	3.607	78.539	0.0811	0.748	25.200	131.508	0.0957

Supplementary Table 2 | Summary of conductivity of the GAP-doped p-type OSCs. Values represent the mean ± SD obtained from eight independent samples.

OSC	IP (eV)[a]	Conductivity ($S\ cm^{-1}$)	
		Pristine film	GAP doped film
P($g_4$2T-T)	4.41	$(3.52 \pm 0.52) \times 10^{-4}$	$(1.91 \pm 0.12) \times 10^{3}$
PgBTTT	4.47	$(2.53 \pm 0.65) \times 10^{-4}$	$(2.73 \pm 0.88) \times 10^{2}$
P3HT	4.96	$(7.65 \pm 2.84) \times 10^{-6}$	$(4.10 \pm 0.80) \times 10^{1}$
PBTTT	5.00	$(1.65 \pm 0.64) \times 10^{-5}$	$(9.65 \pm 1.23) \times 10^{2}$
PTQ1	5.22	$(6.63 \pm 2.28) \times 10^{-7}$	$(3.15 \pm 0.38) \times 10^{0}$
IDTBT	5.38	$(3.57 \pm 1.67) \times 10^{-7}$	$(3.31 \pm 0.21) \times 10^{0}$
PBDB-T-2F	5.47	$(8.75 \pm 0.82) \times 10^{-7}$	$(2.93 \pm 0.55) \times 10^{-1}$
PTB7-Th	5.49	$(5.28 \pm 1.46) \times 10^{-7}$	$(1.81 \pm 0.22) \times 10^{0}$
F8BT	5.90	$(1.13 \pm 0.32) \times 10^{-7}$	$(5.75 \pm 0.59) \times 10^{-6}$

[a]IP values obtained from cyclic voltammetry measurements in Supplementary Fig. 15.

Supplementary Table 3 | Key performance metrics of IDTBT OFETs. Values represent the mean ± SD obtained from six devices. Channel width was fixed at 2 mm.

Channel	Channel length (μm)	Mobility ($cm^2\ V^{-1}\ s^{-1}$)	Threshold voltage (V)	I_{on}/I_{off}
Pristine IDTBT	50	0.14 ± 0.02	−(17.36 ± 2.87)	(4.73 ± 0.18) × 10^3
	100	0.13 ± 0.03	−(15.45 ± 5.08)	(1.08 ± 0.25) × 10^4
IDTBT doped by 1 mM $Na_2S_2O_8$ for 4 s	50	0.25 ± 0.04	−(9.87 ± 3.78)	(1.24 ± 0.41) × 10^4
	100	0.23 ± 0.02	− (10.52 ± 3.06)	(2.35 ± 0.24) × 10^4
IDTBT doped by 10 mM $Na_2S_2O_8$ for 60 s	50	N.A.	N.A.	3.35 ± 2.00
	100	N.A.	N.A.	4.96 ± 3.61

Supplementary References

1 Francis, C. *et al.* Raman spectroscopy and microscopy of electrochemically and chemically doped high-mobility semiconducting polymers. *J. Mater. Chem. C*, **5**, 6176-6184 (2017).

2 Perevedentsev, A. & Campoy-Quiles, M. Rapid and high-resolution patterning of microstructure and composition in organic semiconductors using 'molecular gates'. *Nat. Commun.*, **11**, 3610 (2020).
